# Gangliosidome of a Human Hippocampus in Temporal Lobe Epilepsy Resolved by High-Resolution Tandem Mass Spectrometry

**DOI:** 10.3390/molecules27134056

**Published:** 2022-06-23

**Authors:** Raluca Ica, Kristina Mlinac-Jerkovic, Katarina Ilic, Tomislav Sajko, Cristian V. A. Munteanu, Alina D. Zamfir, Svjetlana Kalanj-Bognar

**Affiliations:** 1 Department of Condensed Matter, National Institute for Research and Development in Electrochemistry and Condensed Matter, 300224 Timisoara, Romania; raluca.ica@gmail.com (R.I.); alina.zamfir@uav.ro (A.D.Z.); 2 Faculty of Physics, West University of Timisoara, 300223 Timisoara, Romania; 3 Croatian Institute for Brain Research, School of Medicine, University of Zagreb, 10000 Zagreb, Croatia; kristina.mlinac.jerkovic@mef.hr (K.M.-J.); katarina.ilic@kcl.ac.uk (K.I.); 4 BRAIN Centre, Department of Neuroimaging, Institute of Psychiatry, Psychology and Neuroscience (IOPPN), King’s College London, London SE5 9NU, UK; 5 Department of Neurosurgery, University Hospital Sestre Milosrdnice, 10000 Zagreb, Croatia; neurosajko@gmail.com; 6 Institute of Biochemistry of the Romanian Academy, Splaiul Independenței 296, 060031 Bucharest, Romania; cristian.v.a.munteanu@gmail.com

**Keywords:** gangliosides, mass spectrometry, temporal epilepsy, human hippocampus

## Abstract

In this study, we developed a high-resolution tandem mass spectrometry (HR MS) approach to assess presumed changes in gangliosidome of a human hippocampus affected by temporal lobe epilepsy (TLE) in comparison with a normal hippocampus. Gangliosides, membrane glycolipids, are particularly diverse and abundant in the human brain, and participate in ion transport and modulation of neuronal excitability. Changes in structural ganglioside pattern potentially linked to TLE molecular pathogenesis have not been explored in detail. Aiming to characterize TLE-specific gangliosidome, we analyzed the native gangliosides purified from a human hippocampal tissue sample affected by TLE and a control hippocampus using HR MS. Marked differences of ganglioside expression were shown in TLE vs. control, particularly with respect to the sialylation degree of components, discovered as a characteristic feature of TLE. Another major finding is the occurrence of tetrasialofucogangliosides in TLE and species modified by either *O*-acetylation or CH_3_COO^−^. Structural analysis by higher-energy collisional dissociation (HCD) MS/MS gave rise to fragmentation patterns implying that the GQ1b (d18:1/18:0) isomer is specifically associated with TLE. Further investigation in a larger sample is needed in order to confirm the discovery of ganglioside structures specifically expressed in human TLE and to provide information on the probable role of gangliosides in the molecular events underlying seizures.

## 1. Introduction

Gangliosides are sialylated membrane glycosphingolipids, expressed with immense structural diversity and abundance in human central nervous system (CNS) [1,2]. A large number of studies confirmed that specific glycolipidomic patterns of different cell types in CNS is important for the modulation of crucial cellular events involving cell membranes, such as cell proliferation, synaptogenesis, myelination and membrane ion transport [3,4,5]. Ganglioside species which predominate in the mammalian brain are GM1, GD1a, GD1b and GT1b, where they are expressed in a specific regional and temporal manner [6,7,8,9]. The diversity of ganglioside structures provides huge variability of their interactions with other membrane lipids and proteins, influencing membrane dynamics and homeostasis. Thus, the utilization of highly sensitive modern mass spectrometric methods has so far enabled detailed structural characterization of human brain gangliosides, and revealed the presence of novel or modified ganglioside structures viewed as specific pathological, diagnostic markers or potential therapeutic targets [10,11]. Alterations in brain ganglioside composition or metabolism have been found in different neurological and neuropsychiatric conditions and are considered to be involved in complex molecular pathogenesis of neurodegeneration [12,13,14,15]. Certain human diseases such as refractory epilepsy are caused by the deficiency in enzymes involved in ganglioside synthesis [16,17]. Although gangliosides are implicated in modulation of neuronal excitability via interactions with ion transport systems, which is altered during seizures, very few studies refer to changes of brain ganglioside compositional pattern in epilepsy [18]. Regrettably, the potential role of gangliosides in molecular pathogenesis of the most common form of epilepsy in humans, temporal lobe epilepsy (TLE) accompanied by hippocampal sclerosis (HS), has not been explored in detail, mainly because such a project requires both the reliable determination of the ganglioside profile and the molecular structure of the species involved as a prerequisite. Such a profiling demands optimal bioanalytical services and protocols able to detect and characterize individual components in the highly complex TLE ganglioside extract.

Amongst all methods, in the past few years, high-resolution mass spectrometry (HR MS), conducted on instruments incorporating the orbital trap analyzer, has emerged as one of the most powerful and reproducible techniques in targeted and untargeted *omics*-related workflows. Based on the high analysis speed, sensitivity and resolving power, as well as the excellent derived accuracy in mass measurement, this platform is able to provide information on single species in minute amounts of clinical samples and on structures with a biomarker value, without the need of mixture separation prior to the MS screening. In combination with nanoelectrospray ionization (nanoESI) and an efficient fragmentation procedure such as the high energy collision-induced dissociation (HCD), HR MS recently became a technique capable of mapping, identification and structural characterization of major and minor ganglioside components in native extracts from human brain [19,20]. Several recent studies have also revealed the major role of nanoESI HR MS and MS/MS in the discovery of ganglioside and fucoganglioside markers in brain tumors [21], neurodegenerative diseases [22] and genetic disorders affecting the central nervous system [23].

In this context, in the present study, an approach based on HR MS and MS/MS using a nanoESIOrbitrap platform operating in the negative ion mode, was for the first time developed, optimized and applied for the determination of ganglioside expression in human brain tissue samples. Our research was focused on the assessment of the changes in the ganglioside pattern in the adult hippocampus affected by TLE vs. normal hippocampus, on the detection of specific TLE structures and the comprehensive characterization of the TLE-associated gangliosidome.

## 2. Results

### 2.1. High-Performance Thin-Layer Chromatography Detects Minor Changes in Ganglioside Compositional Pattern of Human Hippocampal Tissue in Temporal Epilepsy Compared to Control Tissue

**Figure 1:**
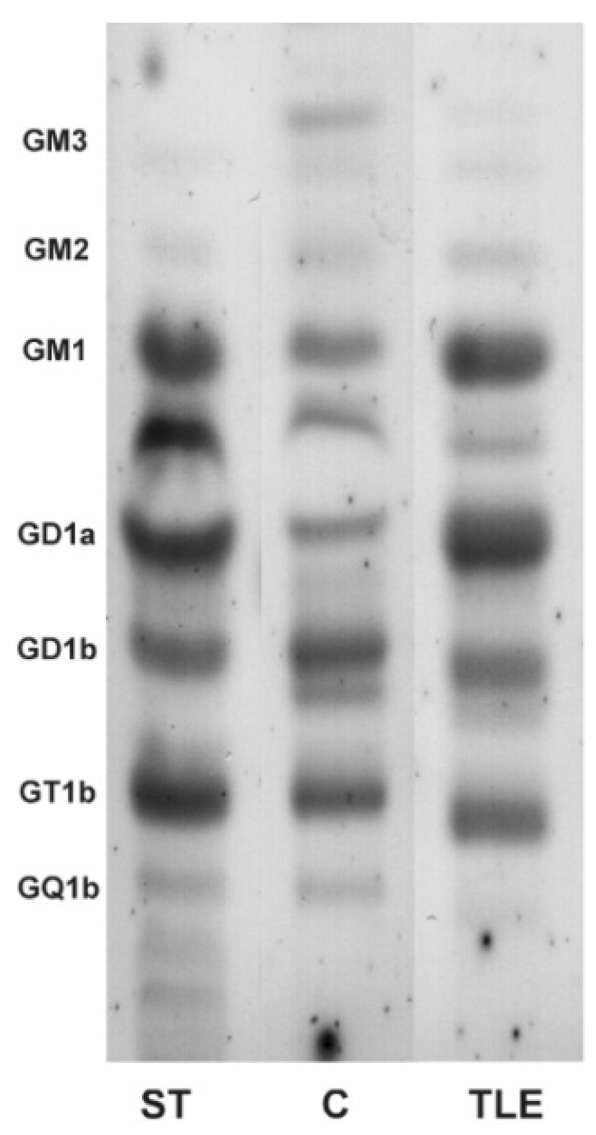
Composition of brain gangliosides isolated from the hippocampus affected by temporal epilepsy (TLE) and control hippocampus (C) was analyzed by high-performance thin-layer chromatography and compared with standard ganglioside mixture (ST).

Qualitative analysis of brain ganglioside composition utilizing high-performance thin-layer chromatography (HPTLC) showed the presence of all major ganglioside fractions in the TLE human hippocampal tissue sample compared with the control (C) hippocampal tissue (Figure 1). No significant compositional change related to GT1b, GD1b, GD1a and GM1 was observed between TLE and C hippocampal tissue. The presence of monosialogangliosides GM2 and GM3, as well as polysialylated GQ1b, was detected in both the pathological and control samples. A higher proportion of the GM1, GD1a and a fraction migrating as GM2 was noticed in TLE vs. C hippocampus.

### 2.2. Comparative Assay by High-Resolution Mass Spectrometry Demonstrates Higher Degree of Gangliosides Species Sialylation, Expression of Fucogangliosides and Modifications of Tri- and Tetrasialogangliosides in Hippocampal Tissue in Temporal Epilepsy

**Figure 2:**
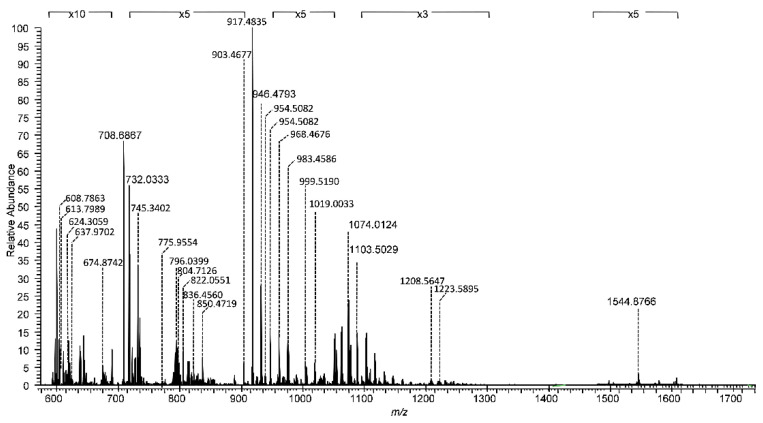
(−) NanoESI HR MS of ganglioside mixture isolated from human hippocampal tissue sample in temporal epilepsy. Sample concentration: 5 pmol·μL^−1^ in MeOH; spray voltage: 0.70 kV; cone voltage: −30 V; acquisition time: 2 min.

**Figure 3:**
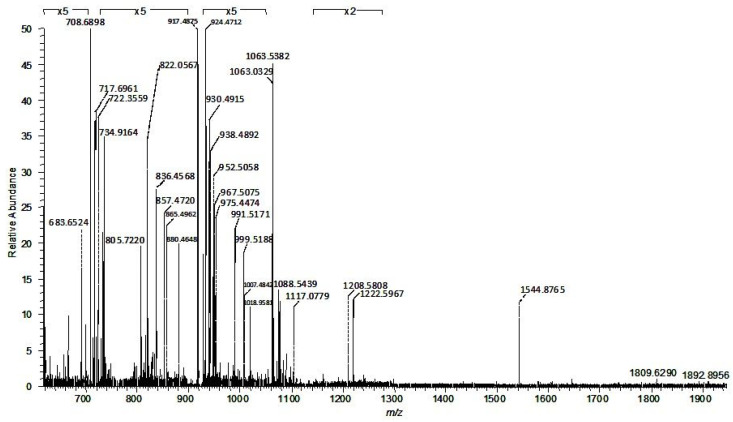
(−) NanoESI HR MS of ganglioside mixture derived from control hippocampal tissue sample. Conditions as in Figure 2.

For nanoESI HR MS comparative screening, 10 µL of the 5 pmol·μL^−1^ hippocampal TLE and control sample solutions in pure methanol were infused by (−) nanoESI into the Orbitrap MS under identical instrumental conditions. The screening mass spectra generated by accumulating scans for 2 min are presented in Figure 2 and Figure 3, while the assignment of the signals, based on the exact mass calculation, is given in Table 1 and Table 2, respectively.

The high resolution of the employed MS platform allowed the ion designation with the excellent average mass accuracy of 5.22 ppm and the standard deviation σ_1_ = 2.29 ppm in the case of TLE and 5.24 ppm with the standard deviation σ_2_ = 1.927 ppm for C hippocampal tissue. Both mass spectra feature a high *signal-to-noise* ratio and a complex molecular ion pattern displaying multicharged molecules (see Supplementary Materials), both of which document not only the presence of an elevated number of structures, but also a diversity of the ceramide composition for many of the identified ganglioside classes. Moreover, the data systematized in Table 1 and Table 2 indicate that the optimized nanoESI HR MS conditions for brain ganglioside profiling provided a fair ionization/detection of major and minor components in the analyzed samples and, most importantly, favored the multicharging of the molecules, which allowed the detection of long-chain polysialylated structures belonging to GT and GQ classes. In Table 1, the average mass accuracy for GT and GQ species is 4.69 and 4.8 ppm, respectively, whereas in Table 2 the average mass accuracy for GT and GQ species is 5.07 and 6.2 ppm, respectively. The mass measurement accuracy of the Orbitrap instrument stated by the producer falls in the 1–5 ppm range; however, we have noticed here and before [19,20,21,23] that the mass tolerance for ganglioside identification is situated in the 0.5–12 ppm range. Our findings are in agreement with previous research reporting differences between the accuracy range indicated by the producer and the optimal mass tolerance for ion identification in the case of large molecules [24].

The inspection of the spectra in Figure 2 and Figure 3 also demonstrate that due to the mild nanoESI screening parameters, at the low spray voltage of 0.70 kV and the low cone voltage of −30 V, the *in-source* fragmentation of the labile modifications of glycan nature such as Neu5Ac and Fuc or non-carbohydrate type such as *O*-Ac and CH_3_COO^−^ were hindered. The anions at *m/z_monisotopic_* 308.1059 and 290.0954 (Neu5Ac), 163.0684 and 145.0579 (Fuc), typical for the *in-source* detachment of the labile Neu5Ac or Fuc, were not detected in the screening mass spectra.

In this comparative assay, the high resolution and accurate mass measurement allowed the discrimination and assignment in TLE hippocampal sample of no less than 105 ions corresponding to 99 ganglioside species differing in the composition of their glycan core and lipid moiety (Table 1) and of 91 ions attributed to 75 distinct gangliosides in the control mixture (Table 2).

Unlike the control sample, TLE hippocampus was found more enriched in polysialylated gangliosides, in particular the tetrasialogangliosides and fucogangliosides. Overall, of the 99 individual components identified in TLE, 87% were found to contain at least two Neu5Ac residues in the structure of the oligosaccharide chain, with an expression in the following ascending order of the number of the detected structures: GT2 < GQ2 < GD2 = GD3 < GT3 < GT1 < GD1 < GQ1.

Obviously, in the TLE hippocampal sample, the highest number of species belonging to the same class is 26 and corresponds to the tetrasialylated tetraoses GQ1 bearing different ceramides and/or modifications of the main oligosaccharide chain by *O*-fucosylation, *O*-acetylation or CH_3_COO^−^. Hence, no less than 26 GQ1 structures were discovered in TLE vs. only 15 in C hippocampal tissue sample. Another important aspect related to the sialylation status of both native ganglioside extracts is related to GQ2 class, which was only discovered in TLE hippocampal sample represented by the species GQ2(d18:1/17:0) and GQ2(d18:1/17:1). Both structures were detected as [M-3H^+^]^3−^ ions of fair relative abundances. The MS data show that, except for the 28 species in the GQ class of which 26 GQ1 and 2 GQ2, no less than 27 trisialylated ganglioside components belonging to the GT class were discovered in the TLE mixture. These species, which have 20 GT1, 1 GT2 and 6 GT3 glycoforms, were discovered here for the first time in association to TLE, while GQ2 species were only detected in TLE. The elevated expression of GQ and GT in TLE hippocampus as compared to C demonstrates that TLE gangliosidome is characterized by a much higher overall sialylation degree of components than the normal hippocampus.

**Figure 4:**
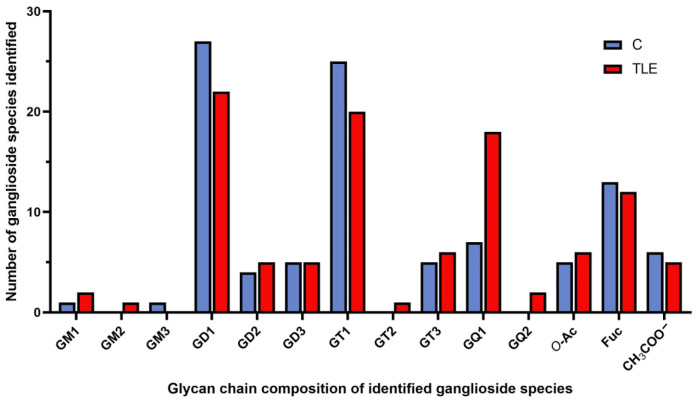
The distribution of the ganglioside species in TLE and control hippocampal tissue samples vs. their glycan chain composition.

A systematic view of the ganglioside pattern in TLE and C hippocampal samples is provided in Figure 4. The histogram comparatively plots the number of species identified vs. the glycan chain composition, which includes the saccharide chain length and the number of sialic acids, counting also those exhibiting labile carbohydrate and non-carbohydrate-type modifications of the main chain such as *O*-Fuc, *O*-Ac and CH_3_COO^−^.

Another characteristic of TLE hippocampal native extract is the occurrence of fucogangliosides and compounds having the oligosaccharide chain modified by either *O*-acetylation or CH_3_COO^−^. Altogether, 25 modified structures were discovered, among which the most interesting are the tri- and tetrasialylated components, whose rather complex glycan chains are supplementary decorated by attachments such as: Fuc-GT3(t18:1/16:1), Fuc-GT3(d18:1/20:0), Fuc-GT3(t18:1/18:0), *O*-Ac-GT1(d18:1/22:0), *O*-Ac-GT1(d18:0/22:0) and, in particular, *O*-Ac-GQ1(d18:1/18:0) identified as a triply deprotonated molecule at *m/z* 819.3877, *O*-Ac-GQ1(t18:1/18:1) also as a [M − 3H^+^]^3−^ signal at *m/z* 824.0505 and (CH_3_COO^−^) GQ1(t18:1/18:3), and a triply charged species at *m/z* 828.7063.

### 2.3. Structural Analysis of GQ1 Species by High-Energy Collision-Induced Dissociation MS/MS Reveals Specific Association of GQ1b Isomer in Hippocampus with Temporal Epilepsy

Since HR MS screening has indicated that the structures in the GQ1 class are strongly expressed in the TLE hippocampal sample, this ganglioside extract numerically dominates and may be a characteristic molecular feature of TLE, in the final stage of research we have conducted a detailed structural analysis by HCD MS/MS, using the [M − 4H^+^]^4−^ detected at *m/z* 603.7844 as the precursor ion. According to the exact mass calculation of the tetradeprotonated glycoform (*m/z_theor_* 603.7830), this ion was assigned with the excellent mass accuracy of 2.32 ppm to the tetrasialotetraose GQ1(d18:1/18:0) species.

**Figure 5:**
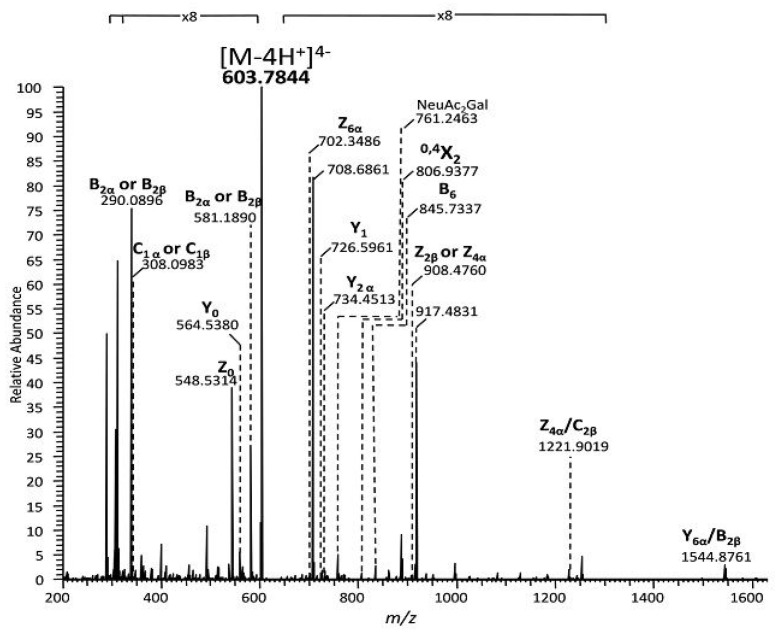
(−) NanoESI HR HCD MS/MS of the [M − 4H^+^]^4−^ detected at *m/z* 603.7844 assigned according to mass calculation to the tetrasialotetraose GQ1(d18:1/18:0). The mass spectrum is a sum of the scans acquired over the entire E_lab_ collision energy range of (35–80) eV, using stepped HCD method; acquisition time: 2 min; other conditions as in Figure 2.

**Figure 6:**
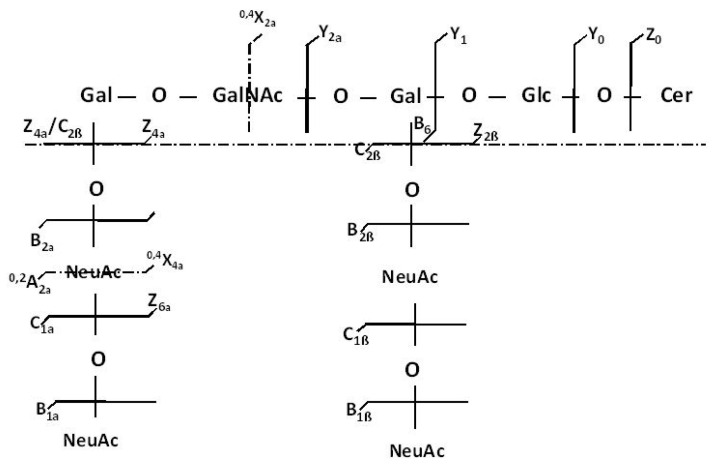
Fragmentation scheme by HCD MS/MS of the [M − 4H^+^]^4−^ detected at *m/z* 603.7844 and the generated product ions.

For the HCD MS/MS fragmentation analysis, the [M − 4H^+^]^4−^ precursor ion was isolated in a window of 1 *m/z* width and subjected to sequencing under variable collision energy in the (35–80) eV range of the applied E_lab_ to make possible the generation of diagnostic fragment ions by both glycosidic bond and cross-ring cleavages. The obtained tandem mass spectrum, generated by summing up the scans accumulated for 2 min across the set range of collision energies, is presented in Figure 5 while the scheme of the fragmentation experienced by this ion under the employed tandem MS conditions, as deduced from the assignment of the fragment ions, is depicted in Figure 6, in a representation correlated to the b isomer.

The inspection of the spectrum in Figure 5 reveals that the optimized HCD experiment gave rise to an important number of singly and multicharged diagnostic fragment ions that not only corroborate the composition of the glycan chain, the ceramide moiety and the overall degree of sialylation, but also the supply information from which the position of the four Neu5Ac residues and the type of the structural isomer may be postulated.

A number of monodeprotonated sequence ions such as Y_1_ at *m/z* 726.5961 corresponding to Glc-Cer, Z_0_ at *m/z* 548.5314 and Y_0_^−^ at *m/z* 564.5380 characterize the aglycone and confirm the (d18:1/18:0) composition of the ceramide, whereas the Z_4α_, Z_6α_ and Z_2β_ ions and particularly the double cleavage [Z_4α_/C_2β_]^−^ at *m/z* 1221.9019 support the Gal-GalNAc-Gal-Glc structure of the glycan backbone and the Gal-GalNAc-Gal-Glc-Cer(d18:1/18:0) structural motif in the fragmented species.

The presence of Neu5Ac at the non-reducing end of the molecule is certified by the high intensity signal at *m/z* 290.0896 associated to the cleavage of the labile Neu5Ac residue; this event led to the generation of the B_1α_ and/or B_1β_, together with their singly charged C_1α_ and/or C_1β_ counterparts at *m/z* 308.0983. Of high importance is the detection of the monocharged fragment ion at *m/z* 581.1890. According to mass calculation, this signal corresponds to the cleaved disialo-Neu5Ac_2_ element, that might originate as either B_2α_ or B_2β_, giving strong evidence on the existence of Neu5Ac–Neu5Ac linkage.

In relation to the type of GQ1 isomer, of structural significance are: (a) the Y_2α_ corresponding to Gal(Neu5Ac_2_)-Glc-Cer and (b) the cross ring cleavage ion ^0,4^X_2α_, which occurred following a 2,4 ring fragmentation of GalNAc monosacharide discovered in a GalNAc-Gal(Neu5Ac_2_)-Glc-Cer motif. Both Y_2α_ and ^0,4^X_2α_ document the linkage of the disialo element to the inner galactose and, together with ^0,4^X_4α_, substantiate the architecture of a ganglioside species having the inner and outer Gal linked to Neu5Ac–Neu5Ac disaccharide. Such a structural pattern is consistent with the GQ1b isomer. Since a few other fragment ions could arise from either GQ1a, GQ1c, or GQ1d isomers, which are known to be expressed in human brain tissue, the presence in the TLE hippocampal sample of these species cannot be excluded either. However, the tandem MS data evidenced here, for the first time, the occurrence of the GQ1b isomer as a characteristic feature of the TLE gangliosidome.

## 3. Discussion

In this study, the gangliosidome of the human hippocampus affected by temporal lobe epilepsy was investigated for the first time by high-resolution tandem mass spectrometry in a comparative assay with a normal hippocampus. The objective of this work was the determination of ganglioside expression in TLE hippocampus by HR MS/MS and the discovery and structural analysis of the disease-associated species. In a previous report [25] on the mapping of the human hippocampus-associated gangliosides in fetal and adult tissues by a hybrid quadrupole time-of-flight (QTOF) mass spectrometer achieving a resolution of 5000 (*m/z* 400), 17 ganglioside components differing in their glycan and ceramide composition were detected. Of these, only four exhibited modifications: three by fucosyl and one by GalNAc attachment. Moreover, a single GQ1 species was found in fetal tissue. In the case of adult hippocampus, the MS screening at 5000 (*m/z* 400) resolution has evidenced only 15 distinct species; the highest sialylation degree determined in the case of adult tissue was three, as documented by the five tetraoses distinguished by QTOF MS: GT1 (d18:1/18:0), GT1 (d18:1/18:1), GT1 (d18:1/20:0), GT1 (d18:1/24:1), and Fuc-GT1 (d18:1/17:0).

In contrast to these results, the 100,000 (*m/z* 400) resolution employed in the present experiments enabled the detection and identification of no less than 99 different gangliosides in hippocampus affected by temporal lobe epilepsy and 75 in the normal adult hippocampus specimen, which represent five times more structures than ever reported in the adult tissue and the highest number of species identified in the hippocampus solely on the basis of MS profiling.

The high resolution and precision in the determination of the masses of the fragment ions and the optimal sequencing conditions in HCD MS/MS provided reliable data on the intimate structure of the tetrasialotetraose GQ1(d18:1/18:0), a ganglioside species specifically expressed in the TLE hippocampus. The fragmentation pathway of this species was found to be consistent with the GQ1b isomer, a configuration displaying one of the disialo elements linked to the inner galactose and one to the outer galactose.

In addition to the high resolution, mass accuracy, reproducibility and fragmentation efficiency, another technical aspect of utmost importance in the case of clinical samples was noticed. The spectra in Figure 2, Figure 3 and Figure 5 were each acquired for 2 min. Considering that the nanoESI flow rate is 500 nL·min^−1^ and the sample concentration 5 pmol·μL^−1^ 2 min of signal acquisition is equivalent to only 5 pmol sample consumption for a mass spectrum, which situates the analysis sensitivity in the low picomole range.

**Figure 7:**
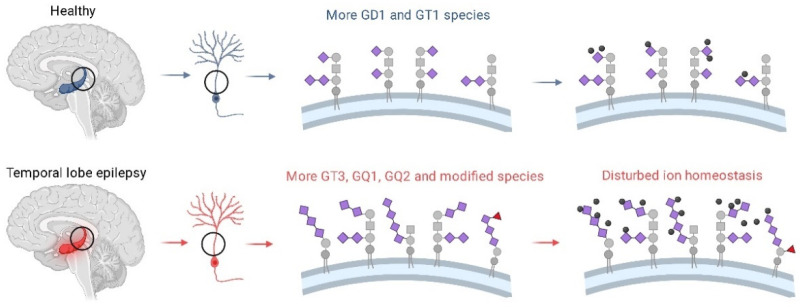
Schematic representation of the presumed relationship between a higher degree of sialylation, ganglioside species modifications and disturbed ion homeostasis occurring in temporal lobe epilepsy.

Finally, a strikingly different ganglioside structural composition of TLE vs. normal hippocampus, detected in this study by utilizing a combination of highly sensitive MS techniques, needs to be further discussed in the context of the potential involvement of gangliosides in TLE molecular pathogenesis. Even though a rather small number of studies are dealing with this topic in humans and mouse models, they provide firm evidence on: (a) the association of seizure phenotypes with genetic disorders linked to enzymes required for ganglioside synthesis [16,17,26]; (b) alterations in ganglioside content and composition in brain tissue in epilepsy [18]. Of interest for comparison with our study are data indicating a link of gangliosides GD3 and GQ1b with temporal epilepsy. The reported increased proportion of GD3, a simple ganglioside species characteristic for proliferating cells during development and activated glial cells in the adult brain is expected, given that glial activation participates in brain tissue response to different types of lesions [27]. Interestingly, the evidenced release of GD3 from activated microglia suggests an additional role of GD3 in neuroinflammation, which contributes to TLE pathology [28,29]. The essential roles of gangliosides in the maintenance of physiological membrane functions in brain tissue are further accentuated by the confirmed relationship of either simple gangliosides such as GM1, GM2, GM3, GD2 and GD3 or more complex (polysialylated) ganglioside structures with various neuropathological conditions [9]. For instance, an increased level of polysialylated ganglioside GQ1b has been proposed to aggravate seizures in animal models, which is relevant to the presented results, demonstrating a specific TLE-related GQ1b expression in a human hippocampal tissue sample [30,31]. The effect of GQ1b on neuronal membrane excitability has been well described and is related to membrane ion transport systems via the modulation of Ca^2+^ channels by gangliosides [32]. Recent studies draw particular attention to the regulation of cellular ion homeostasis by gangliosides as one of the most prominent aspect of gangliosides actions [3,33]. Seemingly, gangliosides are implicated in membrane architectural and functional remodeling occurring in a human TLE pathology in much more complex ways than could have been perceived. In the last two decades, there has been a lack of investigations focusing on the physicochemical properties of membrane gangliosides in TLE; thus, the data presented here bring new insight into the astonishing structural diversity of the gangliosidome specifically associated to TLE. We firstly utilized a less sensitive HPTLC technique for the screening of the ganglioside composition and observed a slightly increased proportion of species migrating as GM1, GM2 and GD1a in TLE compared to the control hippocampal sample. A more sensitive high- resolution MS approach confirmed a large diversity and enrichment of species belonging to GM1, GM2 and GD2 in TLE vs. C hippocampal sample; additionally, it detected an increased expression of polysialylated structures (GT2, GQ1, GQ2), occurrence of fucogangliosides, modified tri- and tetrasialogangliosides, and GQ1b as a specific TLE-related ganglioside structure. Based on the previously described role of gangliosides in ion transport across membranes, it may be presumed that a higher degree of sialylation and modifications of gangliosides observed in human TLE hippocampal tissue sample are associated with alterations of membrane ion homeostasis, as illustrated in Figure 7. The question remains whether increased sialylation is a compensatory effect of a pathologically affected tissue, or part of the pathogenic cascade in TLE, whose clinical phenotype is a consequence of disordered neuronal excitability. The finding of the increased expression of fucogangliosides in TLE hippocampus, along with detected additional modifications of gangliosides by acetylation and *O*-acetylation, is particularly compelling as it indicates a probable disruption of regulatory mechanisms involved in ganglioside synthesis and intracellular trafficking in TLE. Of note also is that modified ganglioside structures at the cell surface may greatly influence the variety of cellular processes related to cell–cell interactions and cellular communication. Further investigation of brain cell-type related gangliosidome pattern is certainly needed in order to clarify more closely a relationship between the occurrence of a particular ganglioside species and the accompanying cellular pathological events such as a (micro)glial response to neuronal damage.

The limitation of this study is that a comparative analysis of the composition and structural features of gangliosides was performed in a single hippocampal tissue sample derived from a patient with temporal epilepsy and neuropathologically unaffected control tissue. A larger case-control study is needed in order to confirm specific TLE-related changes of hippocampal gangliosidome, which could serve as TLE biomarkers, and to clarify the involvement of gangliosides in the molecular pathomechanism of TLE-associated neurodegeneration. Initial findings of this study, although based on a limited number of samples, indicate a highly probable link between altered hippocampal gangliosidome and TLE. The hypothesized role of gangliosides in TLE is in line with recently described structural and metabolic perturbations of other classes of membrane lipids, particularly phosphatidylcholine and phosphatidylethanolamine, in human hippocampus affected by TLE [34].

In conclusion, this study, which utilized an accurate, reproducible and efficient high-resolution MS approach, provides detailed data on altered hippocampal gangliosidome in TLE, establishes TLE-specific expression of GQ1b isomer in human hippocampus, and supports the hypothesized relationship of gangliosides with disturbed ion homeostasis underlying seizures pathogenesis in humans. Since in this study the MS/MS data revealed for the first time the presence in TLE of an isomer with a potential biomarker role, further studies are necessary in order to determine all TLE-specific isomers. Such results are achievable by fragmentation analysis of over 100 ions, which certainly requires high-throughput MS/MS. However, although this method was successful in peptide sequencing, it so far has provided limited structural information on complex molecules such as GSLs. For this reason, we plan the development and application to TLE gangliosides of ion mobility mass spectrometry (IMS MS), which is able to separate isomers, isobars and conformers according to their mobility and provide detailed information on the stoichiometry, topology and structure of even minor compounds in a complex mixture of glycoconjugates, in a single run and on a single instrument.

## 4. Materials and Methods

**Brain tissue samples**: Brain tissue samples utilized for isolation and purification of ganglioside were hippocampal tissue samples affected by temporal epilepsy and control hippocampal tissue samples with no neuropathological changes. The patient with temporal epilepsy (female, aged 28 years) had a history of complex partial seizures, and refractory to antiepileptic medication. The brain magnetic resonance imaging showed right sided hippocampal sclerosis, thus selective amygdalohippocampectomy was indicated. Neuropathological examination confirmed the clinical diagnosis. Control hippocampal tissue was part of the human brain tissue collection at Croatian Institute for Brain Research, Zagreb University School of Medicine, and was obtained from routine autopsy of a person who died of causes not related to neurological conditions (male, aged 63 years). All tissue samples were immediately frozen in liquid nitrogen and stored at −80 °C until further analytical procedures. The study was performed in accordance with the Declaration of Helsinki and was approved by ethical commissions of the University hospital Sestre Milosrdnice, Zagreb (EP-7259/17-17), and Zagreb University School of Medicine (641-01/17-02/01).

**Ganglioside purification**: The ganglioside extraction procedure was performed as described previously [35,36,37]. Briefly, brain tissue samples were homogenized in ice-cold distilled water (W) in a Potter–Elvehjemglass-Teflon homogenizer (DeOtto Lab, Zagreb, Croatia). Lipids were extracted using organic solvents chloroform (C):methanol (M) (1:2, by vol.) followed by partition and repartition by adding M and W to a final volume ratio 1:1:0.7 (chloroform was from T.T.T., Sveta Nedjelja, Croatia; methanol from Honeywell Riedel-de Haen, Seelze, Germany). Upper phases were collected, evaporated to dryness and further purified by gel filtration Sephadex-G25 (Sigma-Aldrich, St. Louis, MO, USA) [38].

**Ganglioside quantification and high performance thin layer chromatography (HPTLC):** Quantitative analysis of ganglioside-bound sialic acid content was determined spectrophotometrically as previously described [36,39]. The absorbances of samples and *N*-acetylneuraminic acid (Sigma-Aldrich, St. Louis, MO, USA) used as a standard in a range of known concentrations were determined at 580 nm. The contents of ganglioside-bound sialic acids are expressed as micrograms of ganglioside-bound sialic acids per gram of fresh tissue *w*.*w*. The purified samples were qualitatively analyzed by HPTLC, which was performed as previously described [35,39,40,41]. Purified samples were dissolved in C:M:W (60:30:4.5, by vol.) and the aliquots spotted to the HPTLC plate. Resolved gangliosides were detected by resorcinol–HCl reagent [39].

**Mass spectrometry analysis:** Mass spectrometry analysis was carried out using a LTQ OrbitrapVelosPro^TM^ mass spectrometer from Thermo Fisher Scientific (Bremen, Germany), equipped with an offline nanoESI source ES 259. Ten microliters of each ganglioside mixture dissolved in methanol (Darmstadt, Germany) to the concentration of 5 pmol·μL^−1^, calculated for an average molecular weight of 2000, were loaded into the borosilicate emitters ES380 (Proxeon, Odense, Denmark) and directly infused into the instrument through the offline nanoESI connected to the instrument using the Nanospray Flex Ion Source (Thermo Scientific, Waltham, MA, USA). The electrospray was initiated at a spray voltage of 0.70 kV and a cone voltage of −30 V, yielding a current of 0.08 µA. Prior to nanoESI infusion, the sample solutions were vortexed in a Biosan Multi-Vortex V-32 (Riga, Latvia) at 2000× *g* rpm, followed by centrifugation in a Beckman Coulter Microfuge^®^16 (Beckman Coulter, Pasadena, CA, USA).

The screening and tandem mass spectra were acquired in the negative ion mode under identical conditions for all samples, with no sheath, sweep or auxiliary gas, in the *m/z* range of 200 to 2000. The MS scans were accumulated in the high-resolution mode, with the resolution set to the value of 100,000 (for *m/z* 400). Prior to experiments, the *m/z* scale was externally calibrated with the standard Pierce^®^ ESI Negative Ion Solution (Thermo Scientific, Waltham, MA, USA). Following the calibration, the average mass accuracy was situated within the normal range of an Orbitrap MS instrument.

The mass spectrometer was operated and controlled by the LTQ Tune Plus v2.7 build 1112 SP2 software (Thermo Scientific, Waltham, MA, USA) running under Windows 7, while the MS data acquisition and possessing were achieved using Xcalibur 3.0.63 software (Thermo Scientific, Waltham, MA, USA). The MS/MS experiments were carried out in the LTQ by high energy collision induced dissociation (HCD) in the HCD collision cell, using Helium 5.0 purity at a pressure of 50 psi as the collision gas, with detection in the HR Orbitrap mode. The precursor ions were selected within an isolation width of 1 *m/z* unit. The HCD MS/MS scans were acquired with a resolution of 20,000 using variable collision energies within a (35–80) eV range, laboratory values (E_lab_), to generate a high coverage of fragment ions. The total ion chromatograms and the derived mass spectra were processed using Xcalibur 2.1 software (Thermo Scientific, Waltham, MA, USA), which allows signal extraction, smoothing and subtraction.

For three replicates the in-run and run-to-run reproducibility of the MS data was 100%, while the day-to-day reproducibility was situated between 96% and 98% in terms of the type, number and intensity of the signals as well as their charge state.

For gangliosides assignment, the abbreviation system introduced by Svennerholm [42], together with the recommendations of IUPAC-IUB Commission on Biochemical Nomenclature (IUPAC-IUB 1998) were used. The ion assignment was based on exact mass calculation and the knowledge we have acquired in our previous studies [10,19,21,22,25] on this type of molecules and the biosynthesis pathways of gangliosides. The assignment of the fragment ions generated within the HCD MS/MS fragmentation experiments followed the nomenclature introduced by Domon and Costello [43] and revised by Costello et al. [44].

## Figures and Tables

**Table 1:** The assignment of the ions detected in the spectrum in Figure 2 and the ganglioside species identified in hippocampal sample in temporal epilepsy by (−) nanoESI HR MS screening. d: dihydroxylated sphingoid base; t: trihydroxylated sphingoid base; *O*-Ac: *O*-acetyl; Fuc: fucose.

No.	*m/z_exp_*	*m/z_theor_*	Proposed Structure	Molecular Ion	ppm
1	603.7844	603.783	GQ1(d18:1/18:0)	[M − 4H^+^]^4−^	2.32
2	608.7863	608.783	GQ1(d18:1/20:0)	[M − 4H^+^]^4−^	5.43
3	610.7934	610.7909	GQ1(d18:1/20:0) and/or GQ1(d20:1/18:0)	[M − 4H^+^]^4−^	4.10
4	613.7989	613.8000	GQ1(d18:0/22:0)	[M − H_2_O − 4H^+^]^4−^	1.79
5	614.2888	614.2855	GQ1(t18:1/20:1)	[M − 4H^+^]^4−^	5.37
6	617.8014	617.799	GQ1(d18:1/22:0)	[M − 4H^+^]^4−^	3.89
7	620.8065	620.8076	GQ1(d18:0/24:0)	[M − H_2_O − 4H^+^]^4−^	1.77
8	624.3059	624.3024	GQ1(d18:1/24:1)	[M − 4H^+^]^4−^	5.61
9	637.9702	637.967	Fuc-GT3(t18:1/16:1)	[M − 4H^+^ +Na^+^]^3−^	5.02
10	674.8742	674.8728	GM2(d18:1/16:2)	[M − 2H^+^]^2−^	2.08
11	699.0083	699.0040	GT1(d18:1/16:0)	[M − 3H^+^]^3−^	6.15
12	708.3524	708.3482	GT1(d18:1/18:0)	[M − 3H^+^]^3−^	5.93
13	713.0035	713.008	GT1(t18:1/18:1)	[M − 3H^+^]^3−^	6.31
14	714.3526	714.3512	GT1(t18:0/18:0)	[M − 3H^+^]^3−^	1.96
15	717.6958	717.692	GT1(d18:1/20:0) and/or GT1(d20:1/18:0)	[M − 3H^+^]^3−^	5.30
16	720.8998	720.8964	GD3(d18:1/16:0)	[M − 2H^+^]^2−^	4.72
17	722.355	722.3522	GT1(t18:1/20:1)	[M − 3H^+^]^3−^	3.88
18	726.3673	726.363	GT1(d18:1/22:1)	[M − 3H^+^]^3−^	5.92
19	727.0393	727.036	GT1(d18:1/22:0)	[M − 3H^+^]^3−^	4.54
20	731.699	731.695	GT1(t18:1/22:1)	[M − 3H^+^]^3−^	5.47
21	734.9159	734.9121	GD3(d18:1/18:0)	[M − 2H^+^]^2−^	5.18
22	733.9079	733.9043	GD3(d18:1/18:0)	[M − 2H^+^]^2−^	4.91
23	735.7112	735.7073	*O*-Ac-GT1(d18:0/22:0)	[M − H_2_O − 3H^+^]^3−^	5.31
24	741.0423	741.0389	*O*-Ac-GT1(d18:1/22:0)	[M − 3H^+^]^3−^	4.59
25	745.3402	745.3465	GQ2(d18:1/17:0)	[M − 3H^+^]^3−^	8.46
26	746.0239	746.0184	GQ2(d18:1/17:1)	[M − 3H^+^]^3−^	7.37
27	771.9344	771.9304	GM1(d18:1/18:0)	[M − 2H^+^]^2−^	5.19
28	775.9554	775.9512	GD3 (d18:1/24:1)	[M − 2H^+^]^2−^	5.42
29	776.9585	776.959	GD3(d18:1/24:0)	[M − 2H^+^]^2−^	0.64
30	789.9623	789.9669	GD3(d18:1/26:1)	[M − 2H^+^]^2−^	5.83
31	796.0399	796.036	GQ1(d18:1/16:0)	[M − 3H^+^]^3−^	4.90
32	804.7126	804.708	GQ1(d18:1/18:1)	[M − 3H^+^]^3−^	5.72
33	805.3842	805.3797	GQ1(d18:1/18:0)	[M − 3H^+^]^3−^	5.59
34	808.7044	808.7079	GQ1(d18:1/19:2)	[M − 3H^+^]^3−^	4.33
35	812.7114	812.707	GQ1(d18:1/18:0)	[M − 4H^+^ + Na^+^]^3−^	5.42
36	814.728	814.724	GQ1(d18:1/20:0) and/or GQ1(d20:1/18:0)	[M − 3H^+^]^3−^	4.91
37	819.3877	819.3832	*O*-Ac-GQ1(d18:1/18:0)	[M − 3H^+^]^3−^	5.49
38	820.7048	820.7063	GQ1(d18:0/18:0)	[M − 5H^+^ + 2Na^+^]^3−^	1.83
39	822.0551	822.0508	GQ1(d18:1/20:0) and/or GQ1(d20:1/18:0)	[M − 4H^+^ + Na^+^]^3−^	5.23
40	824.0505	824.0433	*O*-Ac-GQ1(t18:1/18:1)	[M − 3H^+^]^3−^	8.74
41	828.7063	828.7028	(CH_3_COO^−^) GQ1(t18:1/18:3)	[M − 3H^+^]^3−^	4.23
42	830.0556	830.05	GQ1(d18:0/20:0)	[M − 5H^+^ + 2Na^+^]^3−^	6.75
	830.0556	830.0509	GQ1(d18:1/22:2)	[M − 4H^+^ + Na^+^]^3^	5.66
43	831.4135	831.4115	GD2(d18:1/16:2)	[M − 3H^+^ + Na^+^]^2−^	2.41
44	832.7429	832.7391	GQ1(t18:1/24:0)	[M − H_2_O − 3H^+^]^3−^	4.57
45	835.4501	835.4439	GD2(d18:1/18:1)	[M − 2H^+^]^2−^	7.43
46	836.456	836.452	GD2(d18:1/18:0)	[M − 2H^+^]^2−^	4.78
47	846.4407	846.4349	GD2(d18:1/18:1)	[M − 3H^+^ + Na^+^]^2−^	6.86
48	850.4719	850.467	GD2(d18:1/20:0) and/or GD2(d20:1/18:0)	[M − 2H^+^]^2−^	5.76
49	877.4571	877.4532	(CH_3_COO^−^) GD2(d18:1/18:0)	[M − 3H^+^ + Na^+^]^2−^	4.45
50	891.4515	891.4508	GT3(d18:1/18:0)	[M − 3H^+^ + Na^+^]^2−^	0.79
51	903.4677	903.462	GD1(d18:1/16:0)	[M − 2H^+^]^2−^	6.31
53	916.4758	916.4705	GD1(d18:1/18:1)	[M − 2H^+^]^2−^	5.79
54	917.4836	917.478	GD1(d18:1/18:0)	[M − 2H^+^]^2−^	6.11
55	924.4726	924.4678	GD1(t18:1/18:1)	[M − 2H^+^]^2−^	5.19
56	927.4695	927.4613	GD1(d18:1/18:1)	[M − 3H^+^ + Na^+^]^2−^	8.85
57	928.4746	928.473	GD1(d18:1/18:0)	[M − 3H^+^ + Na^+^]^2−^	1.72
58	930.4907	930.486	GD1(d18:1/20:1)	[M − 2H^+^]^2−^	5.05
59	931.4988	931.494	GD1(d18:1/20:0) and/or GD1(d20:1/18:0)	[M − 2H^+^]^2−^	5.16
60	938.4883	938.4834	GD1(t18:1/20:1)	[M − 2H^+^]^2−^	5.22
61	939.4917	939.4912	GD1(t18:1/20:0)	[M − 2H^+^]^2−^	0.53
62	942.4897	942.4848	GD1(d18:1/20:0) and/or GD1(d20:1/18:0)	[M − 3H^+^ + Na^+^]^2−^	5.20
63	944.5055	944.5016	GD1(d18:1/22:1)	[M − 2H^+^]^2−^	4.13
64	945.5143	945.5102	GD1 (d18:1/22:0) or	[M − 2H^+^]^2−^	4.34
65		945.4976	*O*-Ac-GT3(d18:1/24:0)	[M − H_2_O − 3H^+^ +Na^+^]^2−^	17.67
66	946.4793	946.4808	(CH_3_COO^−^) GD1(d18:1/18:1)	[M − H^+^]^2−^	1.59
67	951.5007	951.4912	*O*-Ac-GD1(d18:1/20:1)	[M − 2H^+^]^2−^	9.99
68	954.5082	954.5147	GD1(t18:0/22:0)	[M − 2H^+^]^2−^	6.81
69	957.4667	957.4537	Fuc-GT3(t18:1/16:1)	[M − 3H^+^ + Na^+^]^2−^	13.58
70	958.4738	958.4798	(CH_3_COO^−^) GD1(d18:1/18:0)	[M − 2H^+^ + Na^+^]^2−^	6.26
71	958.5225	958.5173	GD1(d18:1/24:1)	[M − 2H^+^]^2−^	5.43
72	967.4978	967.504	Fuc-GT3(d18:1/20:0)	[M − 2H^+^]^2−^	6.41
73	968.4676	968.4628	(CH_3_COO) GD1(d18:1/18:1)	[M − 4H^+^ + 2Na^+^]^2−^	4.96
74	979.9872	979.9865	GT2(d18:0/16:0)	[M − 3H^+^ + Na^+^]^2−^	0.72
75	983.4586	983.4682	Fuc-GT3(t18:1/18:0)	[M − 4H^+^ + 2Na^+^]^2−^	9.77
76	985.4728	985.4668	Fuc-GD1(d18:1/18:2)	[M − 3H^+^ + Na^+^]^2−^	6.09
77	987.4903	987.4837	Fuc-GD1(d18:1/18:3)	[M − 2H^+^]^2−^	6.69
78	988.4889	988.4915	Fuc-GD1(d18:1/18:2)	[M − 2H^+^]^2−^	2.63
79	989.4889	989.4999	Fuc-GD1(d18:1/18:1)	[M − 2H^+^]^2−^	11.12
80	990.5087	990.5071	Fuc-GD1(d18:1/18:0)	[M − 2H^+^]^2−^	1.62
81	999.519	999.5123	Fuc-GD1(t18:0/18:0)	[M − 2H^+^]^2−^	6.71
82	1007.4932	1007.498	Fuc-(CH_3_COO^−^) GT3(d18:1/20:1)	[M − 2H^+^ + Na^+^]^2−^	4.77
83	1012.4927	1012.4903	Fuc-GD1(d18:1/20:3)	[M − 3H^+^ + Na^+^]^2−^	2.37
84	1017.9457	1017.9555	GT1(d18:1/12:3)	[M − 2H^+^]^2−^	9.64
85	1019.0033	1019.006	Fuc-(CH_3_COO^−^) GD1(d18:1/18:2)	[M − H^+^]^2−^	2.65
86	1033.9827	1033.987	GT1(d18:1/14:1)	[M − 2H^+^]^2−^	4.16
87	1063.0317	1063.026	GT1(d18:1/14:1)	[M − 2H^+^]^2−^	5.36
88	1074.0124	1074.017	GT1(d18:1/18:0)	[M − 3H^+^ + Na^+^]^2−^	4.28
89	1077.0471	1077.042	GT1(d18:1/20:0) and/or GT1(d20:1/18:0	[M − 2H^+^]^2−^	4.74
90	1084.0365	1084.0311	GT1(t18:1/20:1)	[M − 2H^+^]^2−^	4.98
91	1087.0289	1087.026	GT1(d18:1/22:4)	[M − 2H^+^]^2−^	2.67
92	1088.0368	1088.034	GT1(d18:1/22:3)	[M − 2H^+^]^2^	2.57
93	1095.0025	1094.9923	GT1(d18:1/20:3)	[M − 4H^+^ + 2Na^+^]^2−^	9.32
94	1098.0124	1098.0157	GT1(d18:1/20:1)	[M − 4H^+^ + 2Na^+^]^2−^	3.01
95	1103.0093	1103.02	(CH_3_COO^−^) GT1(d18:1/18:1)	[M − 2H^+^ + Na^+^]^2−^	9.70
96	1109.0428	1109.0365	GT1(t18:1/20:0)	[M − 4H^+^ + 2Na^+^]^2−^	5.68
97	1115.0611	1115.056	GT1(d18:1/24:1)	[M − 3H^+^ + Na^+^]^2−^	4.57
98	1117.077	1117.0716	GT1(d18:0/24:0)	[M − 3H^+^ + Na^+^]^2−^	4.83
99	1208.5647	1208.574	GQ1(d18:1/18:0)	[M − 2H^+^]^2^	7.70
100	1218.5552	1218.557	GQ1(d18:1/18:1)	[M − 3H^+^ + Na^+^]^2−^	1.48
101	1219.5696	1219.565	GQ1(d18:1/18:0)	[M − 3H^+^ + Na^+^]^2−^	3.77
102	1223.5895	1223.597	GQ1(d18:0/20:0)	[M − 2H^+^]^2^	6.13
103	1244.577	1244.571	GQ1(d18:1/20:0) and/or GQ1(d20:1/18:0)	[M − 4H^+^ + 2Na^+^]^2−^	4.82
104	1544.8766	1544.869	GM1(d18:1/18:0)	[M − H^+^]^−^	4.92
105	1572.9064	1572.917	GM1(d18:1/20:0)	[M − H^+^]^−^	6.74

**Table 2:** The assignment of the ions detected in the spectrum in Figure 3 and the ganglioside species identified in control hippocampal tissue sample by (−) nanoESI HR MS screening. d: dihydroxylated sphingoid base; t: trihydroxylated sphingoid base: *O*-Ac: *O*-acetyl; Fuc: fucose.

No.	*m/z_exp_*	*m/z_theor_*	Proposed Structure	Molecular Ion	ppm
1	610.7952	610.791	GQ1(d18:1/20:0)	[M − 4H^+^]^4−^	6.89
2	614.2895	614.2855	GQ1(t18:1/20:1)	[M − 4H^+^]^4−^	6.51
3	643.3823	643.3802	GM3(d18:1/24:1)	[M − H_2_O − 4H^+^ + 2Na^+^]^3−^	3.27
4	683.6524	683.657	GT1(d18:1/14:0)	[M − 3H^+^]^3−^	6.73
5	699.0085	699.004	GT1(d18:1/16:0)	[M − 3H^+^]^3−^	6.44
6	702.3481	702.3444	GT1(d18:1/18:0)	[M − H_2_O − 3H^+^]^3−^	5.27
7	708.3528	708.3482	GT1(d18:1/18:0)	[M − 3H^+^]^3−^	6.50
8	714.3559	714.3512	GT1(t18:0/18:0)	[M − 3H^+^]^3−^	6.58
9	717.6961	717.692	GT1(d18:1/20:0) and/or GT1(d20:1/18:0)	[M − 3H^+^]^3−^	5.72
10	720.9006	720.8964	GD3(d18:1/16:0)	[M − 2H^+^]^2−^	5.83
11	722.3559	722.3522	GT1(t18:1/20:1)	[M − 3H^+^]^3−^	5.12
12	727.0396	727.036	GT1(d18:1/22:0)	[M − 3H^+^]^3−^	4.95
13	731.6997	731.695	GT1(t18:1/22:1)	[M − 3H^+^]^3−^	6.43
14	733.9087	733.9043	GD3(d18:1/18:1)	[M − 2H^+^]^2−^	6.00
15	734.9164	734.9121	GD3(d18:1/18:0)	[M − 2H^+^]^2−^	5.86
16	735.6994	735.7073	*O*-Ac-GT1(d18:0/22:0)	[M − H_2_O − 3H^+^]^3−^	10.75
17	748.9321	748.9277	GD3(d18:1/20:0) and/or GD3(d20:1/18:0)	[M − 2H^+^]^2−^	5.88
18	789.964	789.9669	GD3(d18:1/26:1)	[M − 2H^+^]^2−^	3.68
19	804.7145	804.708	GQ1(d18:1/18:1)	[M − 3H^+^]^3−^	8.08
20	805.3848	805.3797	GQ1(d18:1/18:0)	[M − 3H^+^]^3−^	6.34
21	812.7127	812.707	GQ1(d18:1/18:0)	[M − 4H^+^ + Na^+^]^3−^	7.02
22	814.7285	814.724	GQ1(d18:1/20:0) and/or GQ1(d20:1/18:0)	[M − 3H^+^]^3−^	5.53
23	818.7164	818.7113	*O*-Ac-GQ1(d18:1/18:1)	[M − 3H^+^]^3−^	6.23
24	819.3885	819.3832	*O*-Ac-GQ1(d18:1/18:0)	[M − 3H^+^]^3−^	6.47
25	822.0567	822.0508	GQ1(d18:1/20:0) and/or GQ1(d20:1/18:0)	[M − 4H^+^ + Na^+^]^3−^	7.18
26	836.4568	836.452	GD2(d18:1/18:0)	[M − 2H^+^]^2−^	5.74
27	850.4725	850.467	GD2(d18:1/20:0) and/or GD2(d20:1/18:0)	[M − 2H^+^]^2−^	6.47
28	857.472	857.4752	GD2(d18:1/21:0)	[M − 2H^+^]^2^	3.73
29	865.4962	865.4908	GD2(d18:0/22:0)	[M − 2H^+^]^2^	6.24
30	880.4648	880.4597	GT3(t18:0/18:0)	[M − H_2_O − 2H^+^]^2−^	5.80
31	903.4683	903.462	GD1(d18:1/16:0)	[M − 2H^+^]^2−^	6.98
32	916.4763	916.4705	GD1(d18:1/18:1)	[M − 2H^+^]^2−^	6.33
33	917.484	917.478	GD1(d18:1/18:0)	[M − 2H^+^]^2−^	6.54
34	924.4712	924.4678	GD1(t18:1/18:1)	[M − 2H^+^]^2−^	3.68
35	926.489	926.4834	GD1(t18:0/18:0)	[M − 2H^+^]^2−^	6.05
36	930.4915	930.486	GD1(d18:1/20:1)	[M − 2H^+^]^2−^	5.91
37	931.4995	931.494	GD1(d18:1/20:0) and/or GD1(d20:1/18:0)	[M − 2H^+^]^2−^	5.91
38	938.4892	938.4834	GD1(t18:1/20:1)	[M − 2H^+^]^2−^	6.18
39	940.5046	940.499	GD1(t18:0/20:0)	[M − 2H^+^]^2−^	5.96
40	945.5149	945.5102	GD1 (d18:1/22:0)	[M − 2H^+^]^2−^	4.97
41	946.4831	946.4808	(CH_3_COO^−^) GD1(d18:1/18:1)	[M − H^+^]^2−^	2.43
42	951.4943	951.4912	*O*-Ac-GD1(d18:1/20:1)	[M − 2H^+^]^2−^	3.26
43	952.5049	952.499	*O*-Ac-GD1(d18:1/20:0)	[M − 2H^+^]^2−^	6.20
44	955.4321	955.4381	Fuc-GT3(t18:1/16:3)	[M − 3H^+^ + Na^+^]^2−^	6.28
45	960.5486	960.548	GD1 (d18:1/24:0)	[M − 2H^+^]^2−^	0.62
46	967.5075	967.5044	Fuc-GT3(d18:1/20:0)	[M − 2H^+^]^2−^	3.21
47	969.4736	969.4706	(CH_3_COO^−^) GD1(d18:1/18:0)	[M − 3H^+^ + 2Na^+^]^2−^	3.10
48	975.4827	975.4835	(CH_3_COO^−^)_2_ GD1(d18:1/18:2)	[M − 2H^+^]^2−^	0.82
49	985.4627	985.4668	Fuc-GD1(d18:1/16:2)	[M − 3H^+^ + Na^+^]^2−^	4.16
50	987.4895	987.4837	Fuc-GD1(d18:1/18:3)	[M − 2H^+^]^2−^	5.88
51	989.499	989.4999	Fuc-GD1(d18:1/18:1)	[M − 2H^+^]^2−^	0.91
52	990.5132	990.5078	Fuc-GD1(d18:1/18:0)	[M − 2H^+^]^2−^	5.45
53	991.5171	991.515	Fuc-GD1(d18:0/18:0)	[M − 2H^+^]^2−^	2.12
54	992.5197	992.512	Fuc-GT3(d18:1/24:3)	[M − 2H^+^]^2−^	7.76
55	999.5188	999.5123	Fuc-GD1(t18:0/18:0)	[M − 2H^+^]^2−^	6.51
56	1004.522	1004.523	Fuc-GD1(d18:1/20:0)	[M − 2H^+^]^2−^	1.00
57	1007.4842	1007.482	(CH_3_COO^−^) GD1(d18:1/22:1)	[M − 4H^+^ + 3Na^+^]^2−^	2.18
58	1009.5175	1009.512	(CH_3_COO^−^) Fuc-GT3(d18:1/22:2)	[M − 2H^+^]^2−^	5.45
59	1013.4927	1013.496	Fuc-GD1(d18:1/20:2)	[M − 3H^+^ + Na^+^]^2^	3.26
60	1014.4995	1014.506	Fuc-GD1(d18:1/20:1)	[M − 3H^+^ + Na^+^]^2^	6.41
61	1018.9581	1018.96	GT1(d18:1/12:2)	[M − 2H^+^]^2−^	1.87
62	1025.5497	1025.544	Fuc-GD1(d18:1/23:0)	[M − 2H^+^]^2−^	5.56
63	1049.0169	1049.0102	GT1(d18:1/16:0)	[M − 2H^+^]^2−^	6.39
64	1053.9823	1053.9842	GT1(t18:1/16:3)	[M − 2H^+^]^2−^	1.80
65	1060.0079	1060.0010	GT1(d18:1/16:0)	[M − 3H^+^ + Na^+^]^2−^	6.51
66	1062.025	1062.018	GT1(d18:1/18:1)	[M − 2H^+^]^2−^	6.59
67	1063.0329	1063.0260	GT1(d18:1/18:0)	[M − 2H^+^]^2−^	6.49
68	1074.0231	1074.018	GT1(d18:1/20:3)	[M − 2H^+^]^2−^	4.75
69	1077.0478	1077.042	GT1(d18:1/20:0) and/or GT1(d20:1/18:0)	[M − 2H^+^]^2−^	5.39
70	1081.0081	1081.0065	GT1(t18:1/18:1)	[M − 3H^+^ + Na^+^]^2−^	1.48
71	1083.0008	1082.992	GT1(d18:1/18:2)	[M − 4H^+^ + 2Na^+^]^2−^	8.13
72	1084.0375	1084.031	GT1(t18:1/20:1)	[M − 2H^+^]^2−^	6.00
73	1085.0406	1085.039	GT1(t18:1/20:0)	[M − 2H^+^]^2−^	1.47
74	1086.0534	1086.047	GT1(t18:0/20:0)	[M − 2H^+^]^2−^	5.89
75	1088.0382	1088.034	GT1(d18:1/22:3)	[M − 2H^+^]^2−^	3.86
76	1091.0628	1091.0579	GT1(d18:1/22:0)	[M − 2H^+^]^2−^	4.49
77	1095.0281	1095.023	GT1(t18:1/22:4)	[M − 2H^+^]^2−^	4.66
78	1097.0444	1097.039	GT1(t18:1/22:2)	[M − 2H^+^]^2−^	4.92
79	1102.0544	1102.048	GT1(d18:1/22:0)	[M − 3H^+^ + Na^+^]^2−^	5.81
80	1103.0102	1103.02	(CH_3_COO^−^) GT1(d18:1/18:1)	[M − 2H^+^ + Na^+^]^2−^	8.88
81	1109.0438	1109.0390	GT1(t18:1/24:4)	[M − 2H^+^]^2−^	4.33
82	1112.061	1112.0624	GT1(t18:1/24:1)	[M − 2H^+^]^2−^	1.26
83	1114.0818	1114.078	GT1(t18:0/24:0)	[M − 2H^+^]^2−^	3.41
84	1117.0779	1117.0716	GT1(d18:0/24:0)	[M − 3H^+^ + 2Na^+^]^2−^	7.43
85	1208.5808	1208.574	GQ1(d18:1/18:0)	[M − 2H^+^]^2−^	5.63
86	1218.5662	1218.557	GQ1(d18:1/18:1)	[M − 3H^+^ + Na^+^]^2−^	7.55
87	1219.5713	1219.565	GQ1(d18:1/18:0)	[M − 3H^+^ + Na^+^]^2−^	5.17
88	1222.5967	1222.5899	GQ1(d18:1/20:0)	[M − 2H^+^]^2−^	5.56
89	1229.5871	1229.579	*O*-Ac-GQ1(d18:1/18:0)	[M − 2H^+^]^2−^	6.59
90	1244.5784	1244.571	GQ1(d18:1/20:0) and/or GQ1(d20:1/18:0)	[M − 4H^+^ + 2Na^+^]^2−^	5.95
91	1544.8765	1544.869	GM1(d18:1/18:0)	[M − H^+^]^−^	4.86

## Data Availability

Not applicable.

## Supplementary Materials

The following supporting information can be downloaded at: https://www.mdpi.com/article/10.3390/molecules27134056/s1, The isotopic distribution of species, proving the carried charges.

## References

[1] Svennerholm L. (1980). Gangliosides and synaptic transmission. Adv. Exp. Med. Biol..

[2] Schnaar R.L. (2019). The Biology of Gangliosides. Adv. Carbohydr. Chem. Biochem..

[3] Xu Y., Sun J., Yang L., Zhao S., Liu X., Su Y., Zhang J., Zhao M. (2022). Gangliosides play important roles in the nervous system by regulating ion concentrations. Neurochem. Res..

[4] Ledeen R., Wu G. (2018). Gangliosides of the Nervous System. Methods Mol. Biol..

[5] Rahmann H., Probst W., Muhleisen M. (1982). Gangliosides and synaptic transmission. Jpn. J. Exp. Med..

[6] Tettamanti G., Bonali F., Marchesini S., Zambotti V. (1973). A new procedure for the extraction, purification and fractionation of brain gangliosides. Biochim. Biophys. Acta.

[7] Schnaar R.L., Gerardy-Schahn R., Hildebrandt H. (2014). Sialic acids in the brain: Gangliosides and polysialic acid in nervous system development, stability, disease, and regeneration. Physiol. Rev..

[8] Kracun I., Rosner H., Cosovic C., Stavljenic A. (1984). Topographical atlas of the gangliosides of the adult human brain. J. Neurochem..

[9] Sipione S., Monyror J., Galleguillos D., Steinberg N., Kadam V. (2020). Gangliosides in the Brain: Physiology, Pathophysiology and Therapeutic Applications. Front. Neurosci..

[10] Sarbu M., Ica R., Zamfir A.D. (2022). Gangliosides as Biomarkers of Human Brain Diseases: Trends in Discovery and Characterization by High-Performance Mass Spectrometry. Int. J. Mol. Sci..

[11] Huang F., Bailey L.S., Gao T., Jiang W., Yu L., Bennett D.A., Zhao J., Basso K.B., Guo Z. (2022). Analysis and Comparison of Mouse and Human Brain Gangliosides via Two-Stage Matching of MS/MS Spectra. ACS Omega.

[12] Fukami Y., Ariga T., Yamada M., Yuki N. (2017). Brain Gangliosides in Alzheimer’s Disease: Increased Expression of Cholinergic Neuron-Specific Gangliosides. Curr. Alzheimer Res..

[13] Yanagisawa K. (2007). Role of gangliosides in Alzheimer’s disease. Biochim. Biophys. Acta.

[14] Mlinac K., Kalanj Bognar S. (2010). Role of gangliosides in brain aging and neurodegeneration. Transl. Neurosci..

[15] Sandhoff R., Schulze H., Sandhoff K. (2018). Ganglioside Metabolism in Health and Disease. Prog. Mol. Biol. Transl. Sci..

[16] Simpson M.A., Cross H., Proukakis C., Priestman D.A., Neville D.C., Reinkensmeier G., Wang H., Wiznitzer M., Gurtz K., Verganelaki A. (2004). Infantile-onset symptomatic epilepsy syndrome caused by a homozygous loss-of-function mutation of GM3 synthase. Nat. Genet..

[17] Fragaki K., Ait-El-Mkadem S., Chaussenot A., Gire C., Mengual R., Bonesso L., Beneteau M., Ricci J.E., Desquiret-Dumas V., Procaccio V. (2013). Refractory epilepsy and mitochondrial dysfunction due to GM3 synthase deficiency. Eur. J. Hum. Genet..

[18] Yu R.K., Holley J.A., Macala L.J., Spencer D.D. (1987). Ganglioside changes associated with temporal lobe epilepsy in the human hippocampus. Yale J. Biol. Med..

[19] Sarbu M., Dehelean L., Munteanu C., Ica R., Petrescu A.J., Zamfir A.D. (2019). Human caudate nucleus exhibits a highly complex ganglioside pattern as revealed by high-resolution multistage Orbitrap MS. J. Carbohydr. Chem..

[20] Ica R., Petrut A., Munteanu C.V.A., Sarbu M., Vukelic Z., Petrica L., Zamfir A.D. (2020). Orbitrap mass spectrometry for monitoring the ganglioside pattern in human cerebellum development and aging. J. Mass Spectrom..

[21] Ica R., Simulescu A., Sarbu M., Munteanu C.V.A., Vukelic Z., Zamfir A.D. (2020). High resolution mass spectrometry provides novel insights into the ganglioside pattern of brain cavernous hemangioma. Anal. Biochem..

[22] Dehelean L., Sarbu M., Petrut A., Zamfir A.D. (2019). Trends in Glycolipid Biomarker Discovery in Neurodegenerative Disorders by Mass Spectrometry. Adv. Exp. Med. Biol..

[23] Sarbu M., Ica R., Petrut A., Vukelic Z., Munteanu C.V.A., Petrescu A.J., Zamfir A.D. (2019). Gangliosidome of human anencephaly: A high resolution multistage mass spectrometry study. Biochimie.

[24] Yu F., Fang H., Xiao K., Liu Y., Xue B., Tian Z. (2016). Mass measurement accuracy of the Orbitrap in intact proteome analysis. Rapid Commun. Mass Spectrom..

[25] Vukelic Z., Zarei M., Peter-Katalinic J., Zamfir A.D. (2006). Analysis of human hippocampus gangliosides by fully-automated chip-based nanoelectrospray tandem mass spectrometry. J. Chromatogr. A.

[26] Wu G., Lu Z.H., Wang J., Wang Y., Xie X., Meyenhofer M.F., Ledeen R.W. (2005). Enhanced susceptibility to kainate-induced seizures, neuronal apoptosis, and death in mice lacking gangliotetraose gangliosides: Protection with LIGA 20, a membrane-permeant analog of GM1. J. Neurosci..

[27] Goldman J.E., Reynolds R. (1996). A reappraisal of ganglioside GD3 expression in the CNS. Glia.

[28] Simon B.M., Malisan F., Testi R., Nicotera P., Leist M. (2002). Disialoganglioside GD3 is released by microglia and induces oligodendrocyte apoptosis. Cell Death Differ..

[29] Gershen L.D., Zanotti-Fregonara P., Dustin I.H., Liow J.S., Hirvonen J., Kreisl W.C., Jenko K.J., Inati S.K., Fujita M., Morse C.L. (2015). Neuroinflammation in Temporal Lobe Epilepsy Measured Using Positron Emission Tomographic Imaging of Translocator Protein. JAMA Neurol..

[30] Kato K., Iwamori M., Hirabayashi Y. (2008). Increase of GQ1b in the hippocampus of mice following kindled-seizures. Neurosci. Lett..

[31] Nakamura Y., Morimoto K., Okamoto M. (1989). Modification of amygdala kindling by intracerebroventricularly administered gangliosides in rats. Exp. Neurol..

[32] Fujii S., Igarashi K., Sasaki H., Furuse H., Ito K., Kaneko K., Kato H., Inokuchi J., Waki H., Ando S. (2002). Effects of the mono- and tetrasialogangliosides GM1 and GQ1b on ATP-induced long-term potentiation in hippocampal CA1 neurons. Glycobiology.

[33] Ilic K., Lin X., Malci A., Stojanovic M., Puljko B., Rozman M., Vukelic Z., Heffer M., Montag D., Schnaar R.L. (2021). Plasma Membrane Calcium ATPase-Neuroplastin Complexes Are Selectively Stabilized in GM1-Containing Lipid Rafts. Int. J. Mol. Sci..

[34] Ajith A., Mondal S., Chattopadhyay S., Kumar A., Sthanikam Y., Chacko A.G., Prabhu K., Chacko G., Vanjare H.A., Rajesh V. (2021). Mass Spectrometry Imaging Deciphers Dysregulated Lipid Metabolism in the Human Hippocampus Affected by Temporal Lobe Epilepsy. ACS Chem. Neurosci..

[35] Svennerholm L., Fredman P. (1980). A procedure for the quantitative isolation of brain gangliosides. Biochim. Biophys. Acta.

[36] Mlinac K., Fabris D., Vukelic Z., Rozman M., Heffer M., Bognar S.K. (2013). Structural analysis of brain ganglioside acetylation patterns in mice with altered ganglioside biosynthesis. Carbohydr. Res..

[37] Schnaar R.L. (1994). Isolation of glycosphingolipids. Methods Enzymol..

[38] Wells M.A., Dittmer J.C. (1963). The Use of Sephadex for the Removal of Nonlipid Contaminants from Lipid Extracts. Biochemistry.

[39] Svennerholm L. (1957). Quantitative estimation of sialic acids. II. A colorimetric resorcinol-hydrochloric acid method. Biochim. Biophys. Acta.

[40] Mlinac-Jerkovic K., Ilic K., Zjalic M., Mandic D., Debeljak Z., Balog M., Damjanovic V., Macek Hrvat N., Habek N., Kalanj-Bognar S. (2021). Who’s in, who’s out? Re-evaluation of lipid raft residents. J. Neurochem..

[41] Schnaar R.L., Needham L.K. (1994). Thin-layer chromatography of glycosphingolipids. Methods Enzymol..

[42] Svennerholm L. (1980). Ganglioside designation. Adv. Exp. Med. Biol..

[43] Domon B., Costello C.E. (1988). A systematic nomenclature for carbohydrate fragmentations in FAB-MS/MS spectra of glycoconjugates. Glycoconj. J..

[44] Costello C.E., Juhasz P., Perreault H. (1994). New mass spectral approaches to ganglioside structure determinations. Prog. Brain Res..

