# Supplementary material for: Gangliosidome of a Human Hippocampus in Temporal Lobe Epilepsy Resolved by High-Resolution Tandem Mass Spectrometry
Source: Molecules. 2022 Jun 23;27(13):4056. doi: 10.3390/molecules27134056 (PMC9268582; doi:10.3390/molecules27134056)
Supplement: Supplementary file 1 [file molecules-27-04056-s001.zip › molecules-1734363-supplementary.pdf]

Gangliosidome of a Human Hippocampus in Temporal Lobe Epilepsy Resolved by High-Resolution Tandem Mass Spectrometry

Raluca Ica [1,2], Kristina Mlinac-Jerkovic [3], Katarina Ilic [3,4], Tomislav Sajko [5], Cristian V.A. Munteanu [6], Alina D. Zamfir [1,2] and Svjetlana Kalanj-Bognar [3,*]

[1] Department of Condensed Matter, National Institute for Research and Development in Electrochemistry and Condensed Matter, 300224 Timisoara, Romania; raluca.ica@gmail.com (R.I.); alina.zamfir@uav.ro (A.D.Z.)
[2] Faculty of Physics, West University of Timisoara, 300223 Timisoara, Romania
[3] Croatian Institute for Brain Research, School of Medicine, University of Zagreb, 10000 Zagreb, Croatia; kristina.mlinac.jerkovic@mef.hr (K.M.-J.); katarina.ilic@kcl.ac.uk (K.I.)
[4] BRAIN Centre, Department of Neuroimaging, Institute of Psychiatry, Psychology and Neuroscience (IOPPN), King's College London, London SE5 9NU, UK
[5] Department of Neurosurgery, University Hospital Sestre Milosrdnice, 10000 Zagreb, Croatia; neurosajko@gmail.com
[6] Institute of Biochemistry of the Romanian Academy, Splaiul Independenței 296, 060031 Bucharest, Romania; cristian.v.a.munteanu@gmail.com
* Correspondence: svjetlana.kalanj.bognar@mef.hr

Supplementary Materials: The isotopic distribution of species, proving the carried charges.

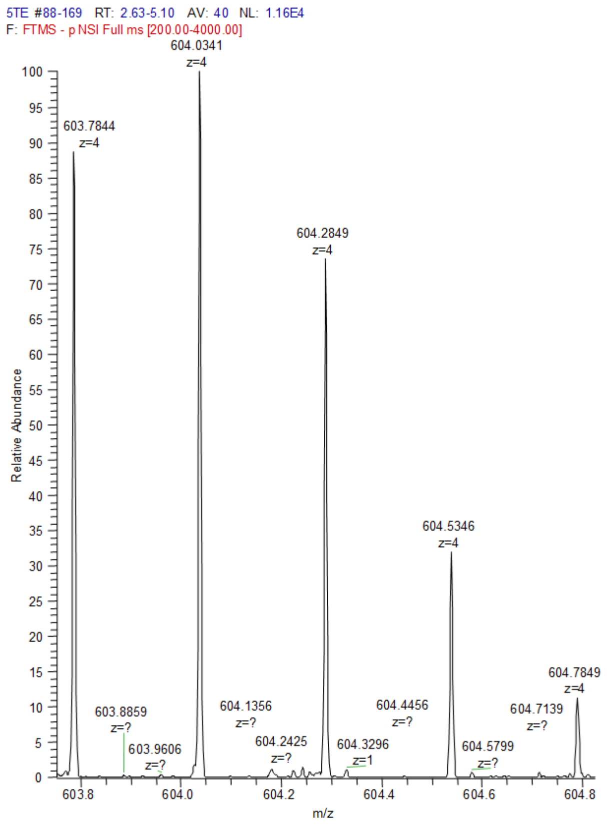
5TE #88-169 RT: 2.63-5.10 AV: 40 NL: 1.16E4
F: FTMS - p NSI Full ms [200.00-4000.00]
604.0341 z=4
603.7844 z=4
604.2849 z=4
604.5346 z=4
604.7849 z=4
603.8859 z=?
603.9606 z=?
604.1356 z=?
604.2425 z=?
604.3296 z=1
604.4456 z=?
604.5799 z=?
604.7139 z=?
Relative Abundance
m/z

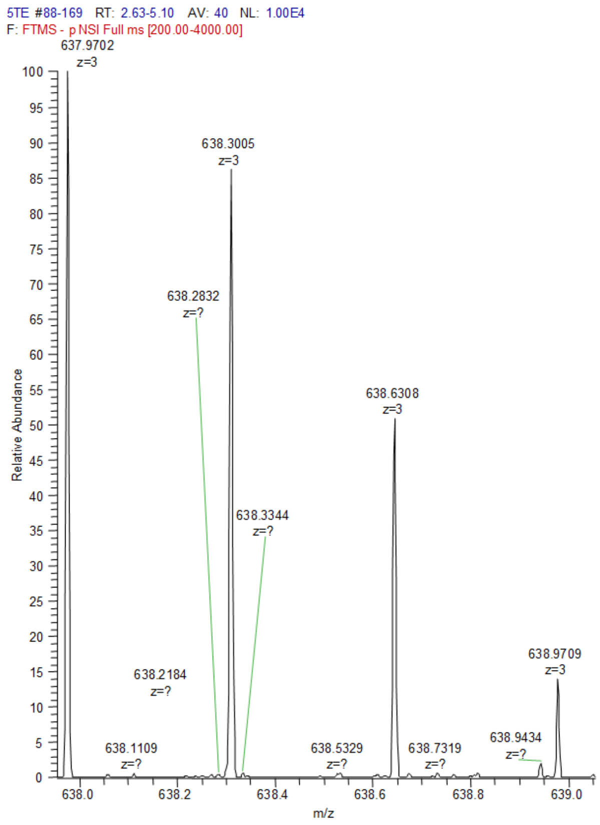
5TE #88-169 RT: 2.63-5.10 AV: 40 NL: 1.00E4
F: FTMS - p NSI Full ms [200.00-4000.00]
637.9702 z=3
638.3005 z=3
638.2832 z=?
638.6308 z=3
638.3344 z=?
638.9709 z=3
638.2184 z=?
638.9434 z=?
638.1109 z=?
638.5329 z=?
638.7319 z=?
Relative Abundance
m/z

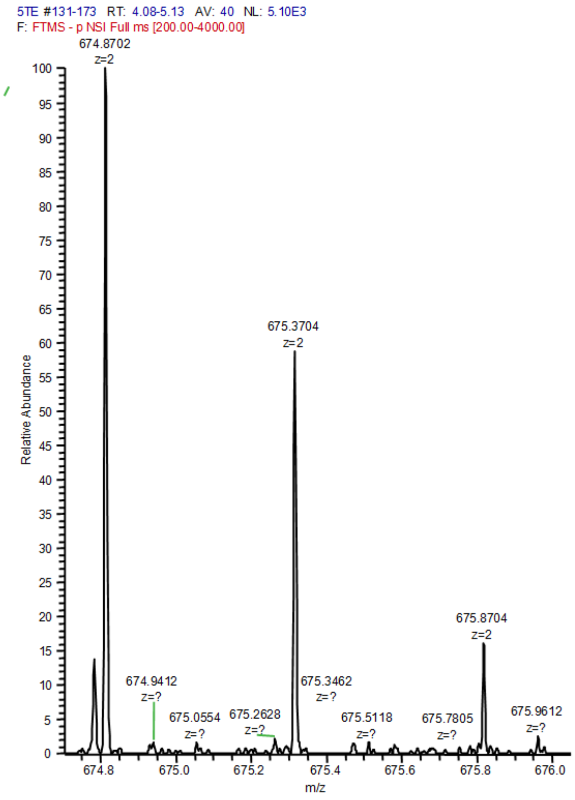
5TE #131-173 RT: 4.08-5.13 AV: 40 NL: 5.10E3
F: FTMS - p NSI Full ms [200.00-4000.00]
674.8702 z=2
675.3704 z=2
675.8704 z=2
674.9412 z=?
675.3462 z=?
675.0554 z=?
675.2628 z=?
675.5118 z=?
675.7805 z=?
675.9612 z=?
Relative Abundance
m/z

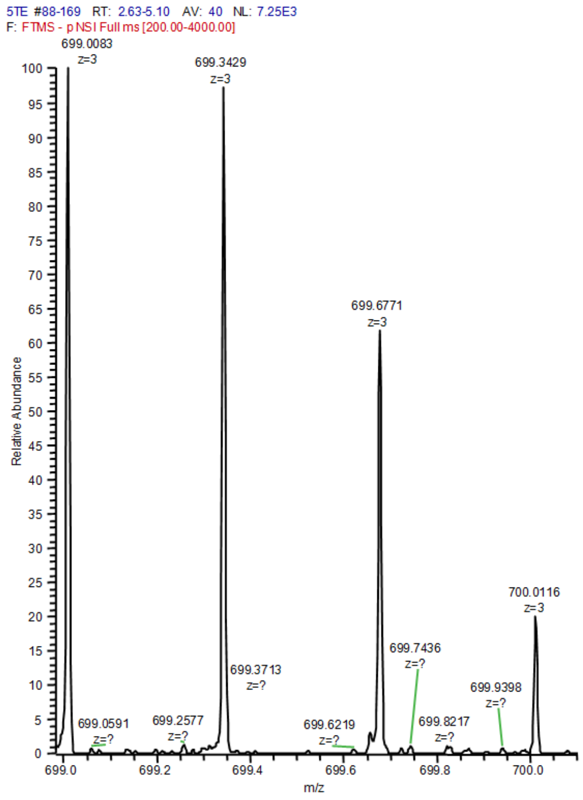
5TE #88-169 RT: 2.63-5.10 AV: 40 NL: 7.25E3
F: FTMS - p NSI Full ms [200.00-4000.00]
699.0083 z=3
699.3429 z=3
699.6771 z=3
700.0116 z=3
699.7436 z=?
699.3713 z=?
699.9398 z=?
699.0591 z=?
699.2577 z=?
699.6219 z=?
699.8217 z=?
Relative Abundance
m/z

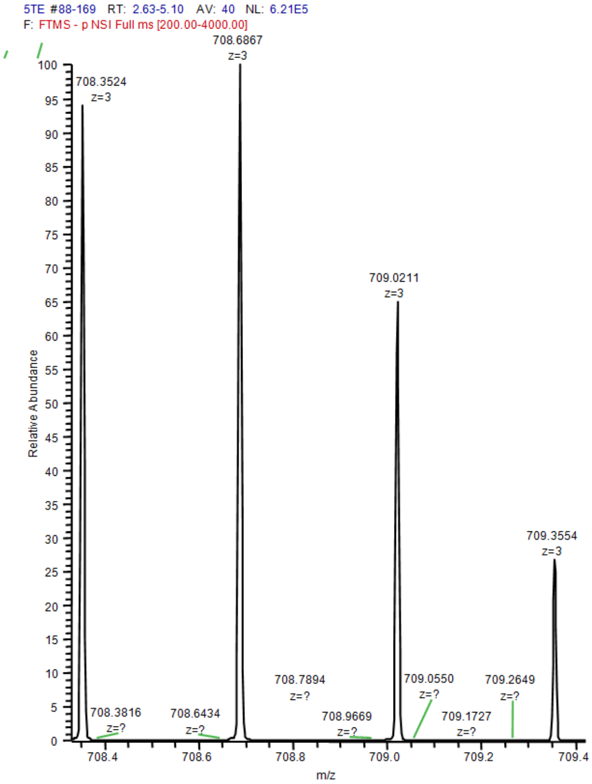
5TE #88-169 RT: 2.63-5.10 AV: 40 NL: 6.21E5
F: FTMS - p NSI Full ms [200.00-4000.00]
708.3524 z=3
708.6867 z=3
709.0211 z=3
709.3554 z=3
708.3816 z=?
708.6434 z=?
708.7894 z=?
708.9669 z=?
709.0550 z=?
709.1727 z=?
709.2649 z=?
Relative Abundance
m/z

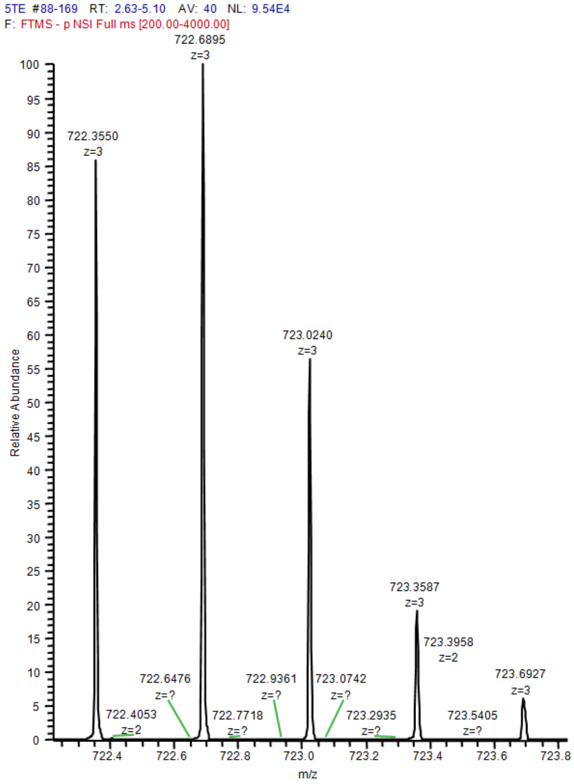
5TE #88-169 RT: 2.63-5.10 AV: 40 NL: 9.54E4
F: FTMS - p NSI Full ms [200.00-4000.00]
722.6895 z=3
722.3550 z=3
723.0240 z=3
723.3587 z=3
723.3958 z=2
723.6927 z=3
722.6476 z=?
722.4053 z=2
722.9361 z=?
722.7718 z=?
723.0742 z=?
723.2935 z=?
723.5405 z=?
Relative Abundance
m/z

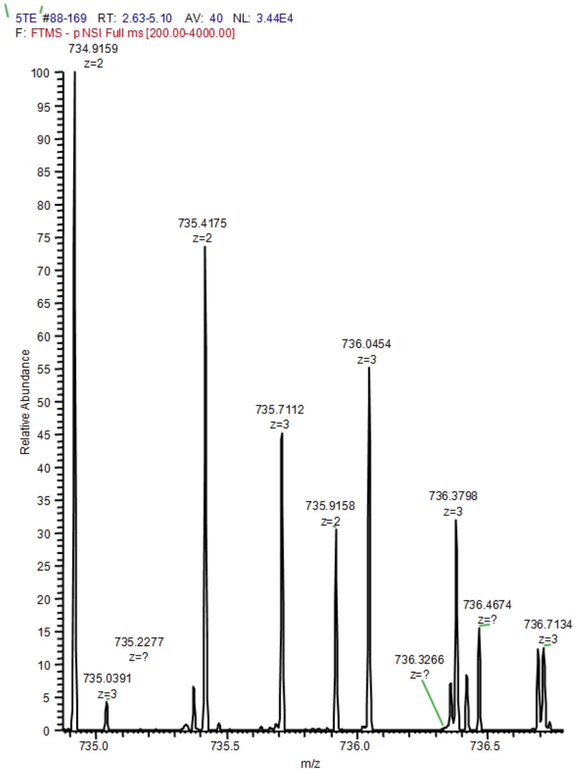
5TE #88-169 RT: 2.63-5.10 AV: 40 NL: 3.44E4
F: FTMS - p NSI Full ms [200.00-4000.00]
734.9159 z=2
735.4175 z=2
736.0454 z=3
735.7112 z=3
735.9158 z=2
736.3798 z=3
736.4674 z=?
736.7134 z=3
735.2277 z=?
736.3266 z=?
735.0391 z=3
Relative Abundance
m/z

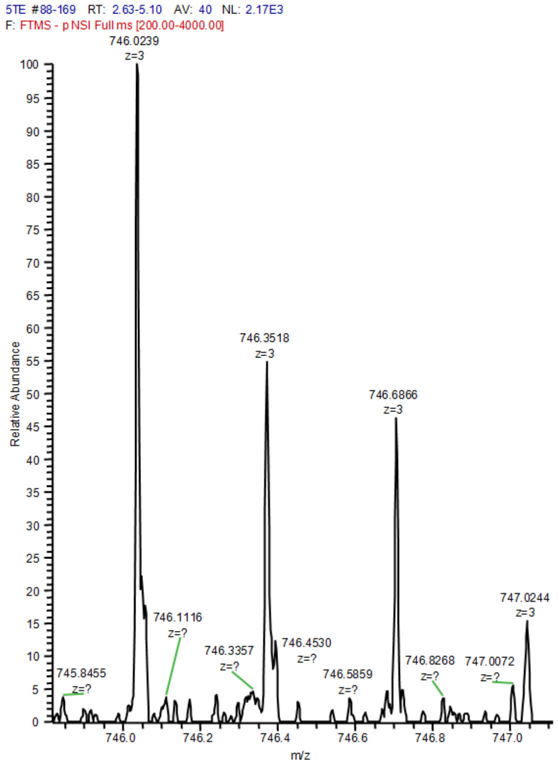
5TE #88-169 RT: 2.63-5.10 AV: 40 NL: 2.17E3
F: FTMS - p NSI Full ms [200.00-4000.00]
746.0239 z=3
746.3518 z=3
746.6866 z=3
747.0244 z=3
746.1116 z=?
746.3357 z=?
746.4530 z=?
746.5859 z=?
746.8268 z=?
747.0072 z=?
745.8455 z=?
Relative Abundance
m/z

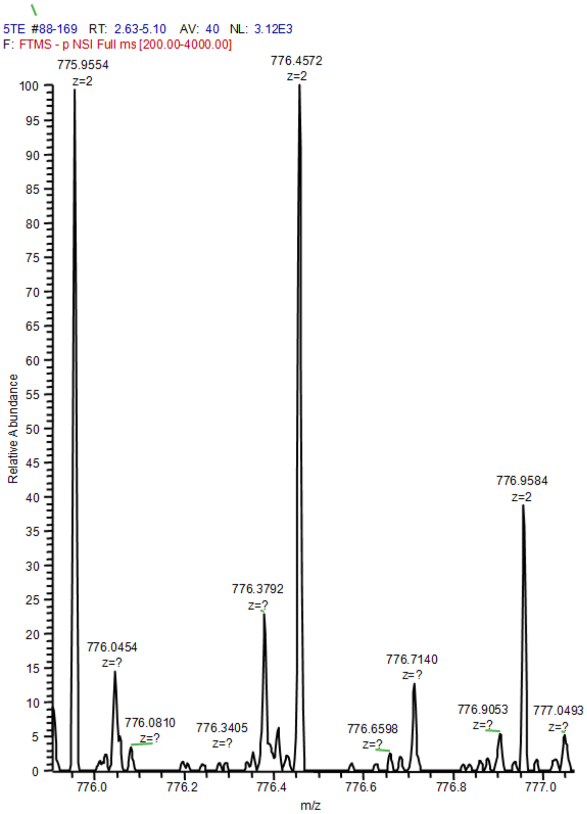
5TE #88-169 RT: 2.63-5.10 AV: 40 NL: 3.12E3
F: FTMS - p NSI Full ms [200.00-4000.00]
775.9554 z=2
776.4572 z=2
776.9584 z=2
776.3792 z=?
776.0454 z=?
776.7140 z=?
776.0810 z=?
776.3405 z=?
776.6598 z=?
776.9053 z=?
777.0493 z=?
Relative Abundance
m/z

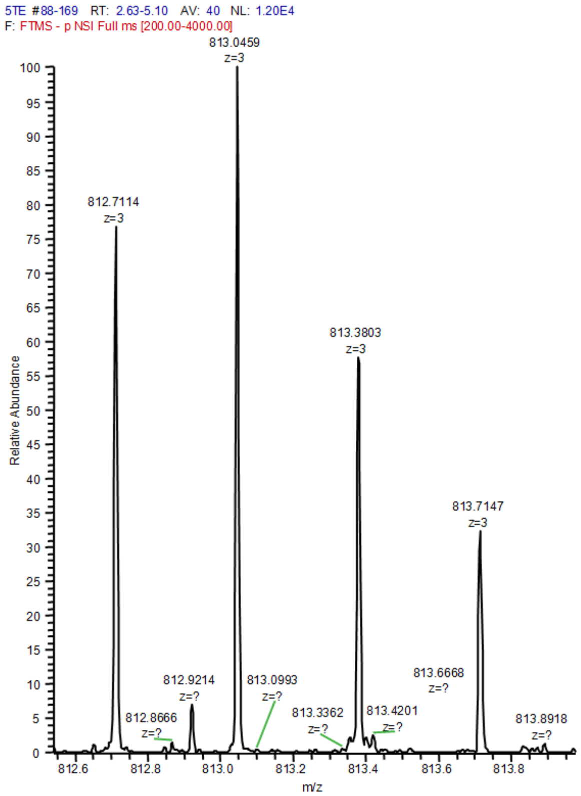
5TE #88-169 RT: 2.63-5.10 AV: 40 NL: 1.20E4
F: FTMS - p NSI Full ms [200.00-4000.00]
813.0459 z=3
812.7114 z=3
813.3803 z=3
813.7147 z=3
812.9214 z=?
813.0993 z=?
813.6668 z=?
812.8666 z=?
813.3362 z=?
813.4201 z=?
813.8918 z=?
Relative Abundance
m/z

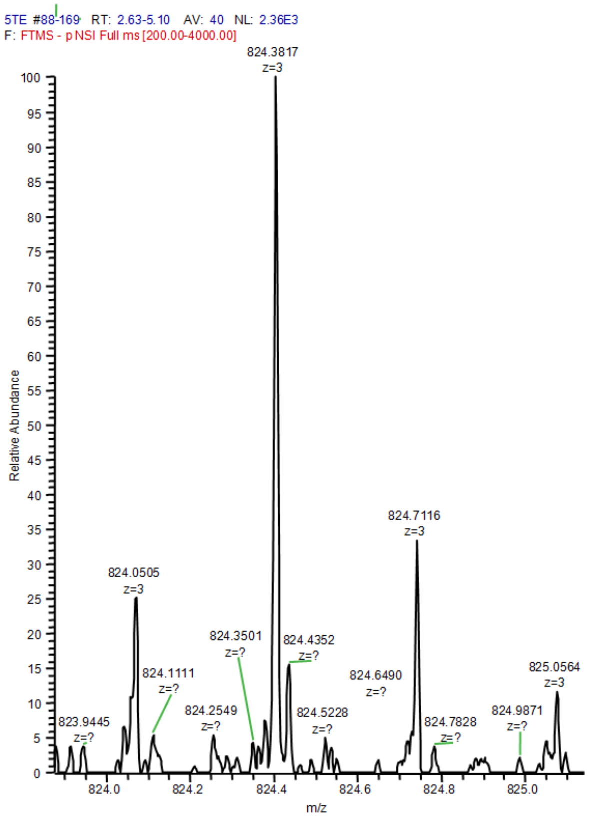
5TE #88-169 RT: 2.63-5.10 AV: 40 NL: 2.36E3
F: FTMS - p NSI Full ms [200.00-4000.00]
824.3817 z=3
824.7116 z=3
824.0505 z=3
824.3501 z=?
824.4352 z=?
824.1111 z=?
825.0564 z=3
824.6490 z=?
823.9445 z=?
824.2549 z=?
824.5228 z=?
824.7828 z=?
824.9871 z=?
Relative Abundance
m/z

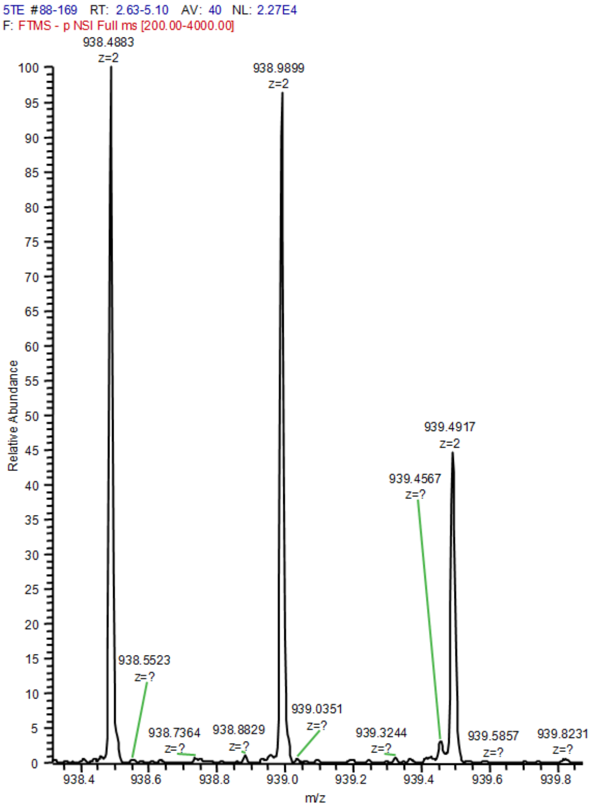
5TE #88-169 RT: 2.63-5.10 AV: 40 NL: 2.27E4
F: FTMS - p NSI Full ms [200.00-4000.00]
938.4883 z=2
938.9899 z=2
939.4917 z=2
939.4567 z=?
938.5523 z=?
939.0351 z=?
938.7364 z=?
938.8829 z=?
939.3244 z=?
939.5857 z=?
939.8231 z=?
Relative Abundance
m/z

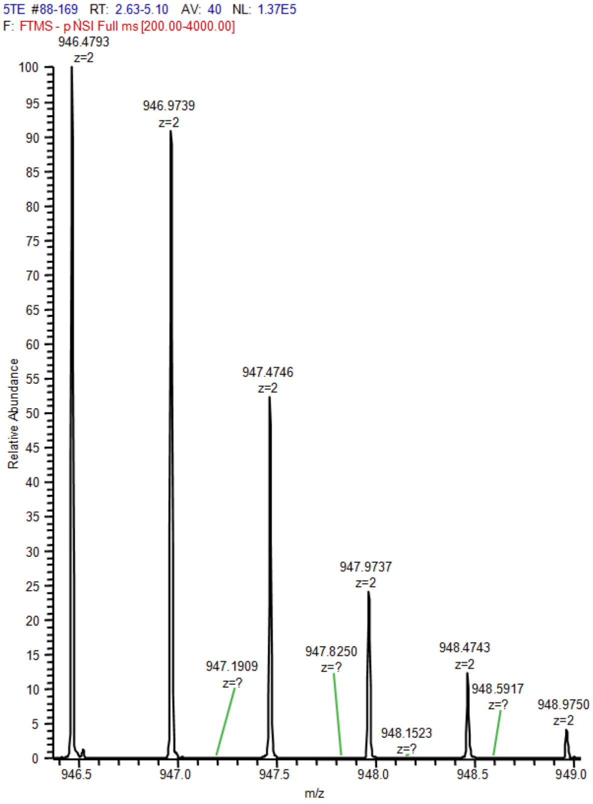
5TE #88-169 RT: 2.63-5.10 AV: 40 NL: 1.37E5
F: FTMS - p NSI Full ms [200.00-4000.00]
946.4793 z=2
946.9739 z=2
947.1909 z=?
947.4746 z=2
947.8250 z=?
947.9737 z=2
948.1523 z=?
948.4743 z=2
948.5917 z=?
948.9750 z=2
Relative Abundance
m/z

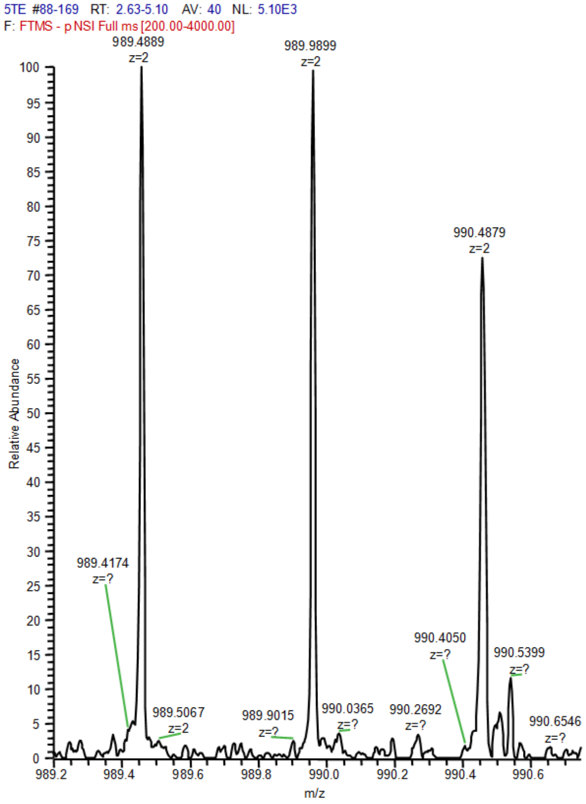
5TE #88-169 RT: 2.63-5.10 AV: 40 NL: 5.10E3
F: FTMS - p NSI Full ms [200.00-4000.00]
989.4889 z=2
989.9899 z=2
990.4879 z=2
989.4174 z=?
989.5067 z=2
989.9015 z=?
990.0365 z=?
990.2692 z=?
990.4050 z=?
990.5399 z=?
990.6546 z=?
Relative Abundance
m/z

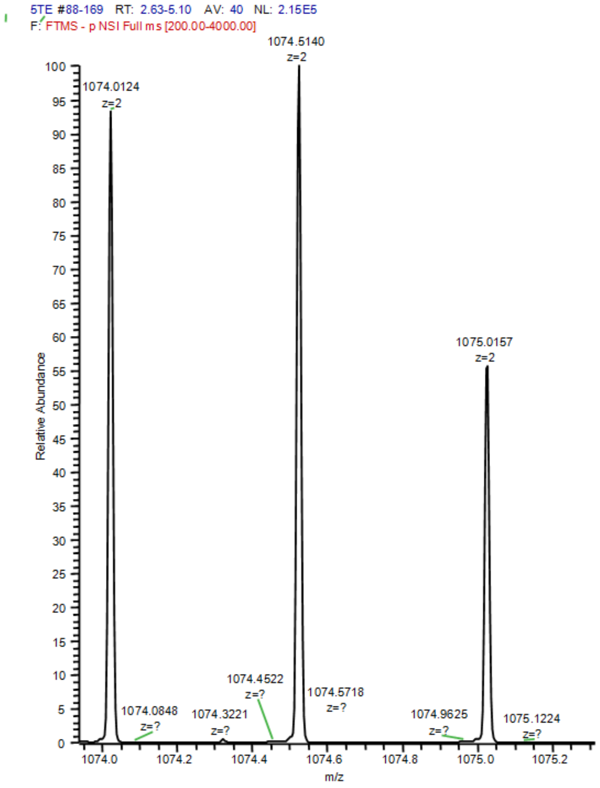
5TE #88-169 RT: 2.63-5.10 AV: 40 NL: 2.15E5
F: FTMS - p NSI Full ms [200.00-4000.00]
1074.5140 z=2
1074.0124 z=2
1075.0157 z=2
1074.0848 z=?
1074.3221 z=?
1074.4522 z=?
1074.5718 z=?
1074.9625 z=?
1075.1224 z=?
Relative Abundance
m/z

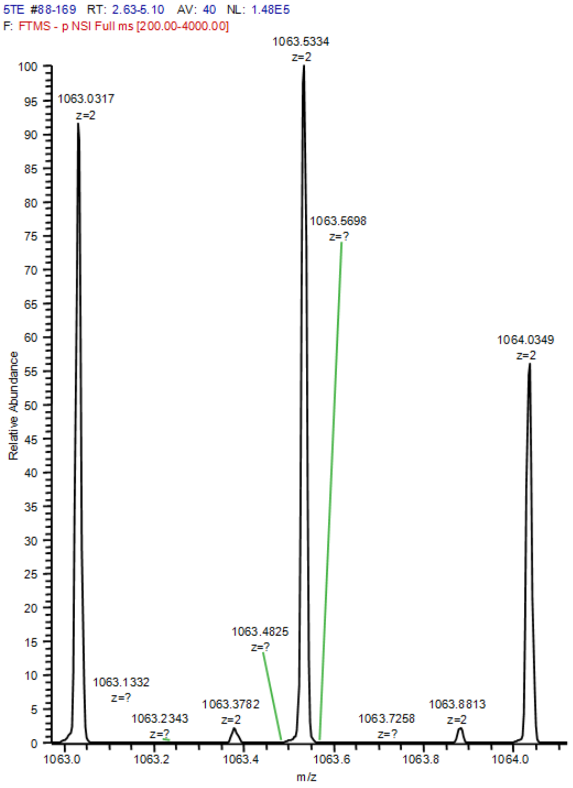
5TE #88-169 RT: 2.63-5.10 AV: 40 NL: 1.48E5
F: FTMS - p NSI Full ms [200.00-4000.00]
1063.5334 z=2
1063.0317 z=2
1063.5698 z=?
1064.0349 z=2
1063.4825 z=?
1063.1332 z=?
1063.2343 z=?
1063.3782 z=2
1063.7258 z=?
1063.8813 z=2
Relative Abundance
m/z

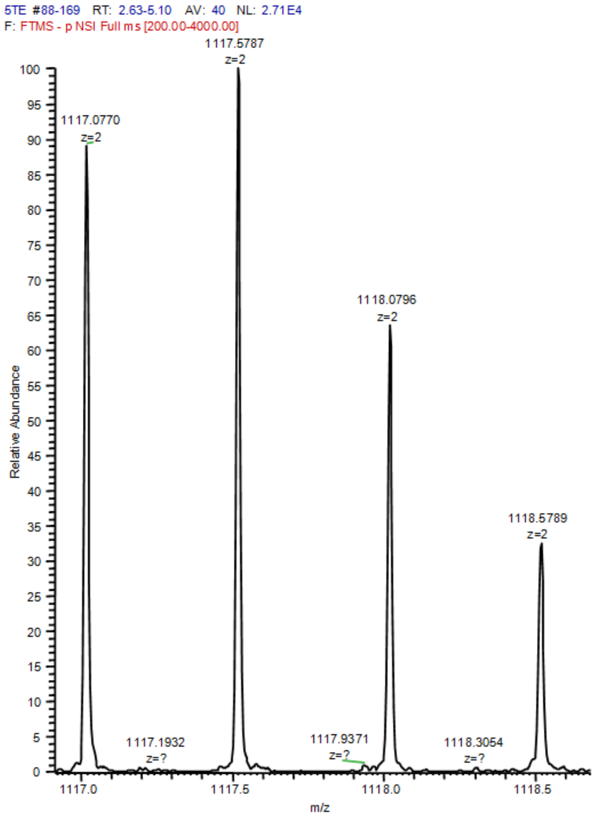
5TE #88-169 RT: 2.63-5.10 AV: 40 NL: 2.71E4
F: FTMS - p NSI Full ms [200.00-4000.00]
1117.0770 z=2
1117.1932 z=?
1117.5787 z=2
1117.9371 z=?
1118.0796 z=2
1118.3054 z=?
1118.5789 z=2
Relative Abundance
m/z

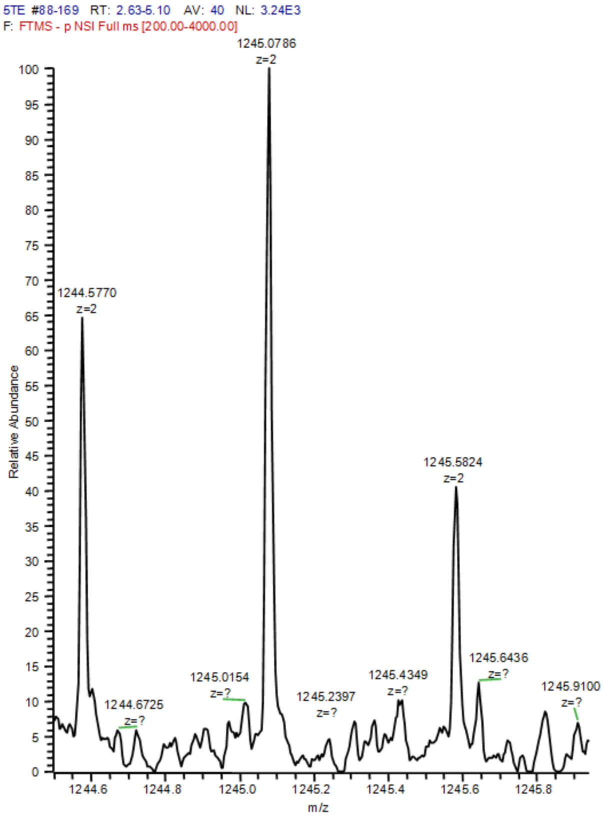
5TE #88-169 RT: 2.63-5.10 AV: 40 NL: 3.24E3
F: FTMS - p NSI Full ms [200.00-4000.00]
1244.5770 z=2
1244.6725 z=?
1245.0154 z=?
1245.0786 z=2
1245.2397 z=?
1245.4349 z=?
1245.5824 z=2
1245.6436 z=?
1245.9100 z=?
Relative Abundance
m/z

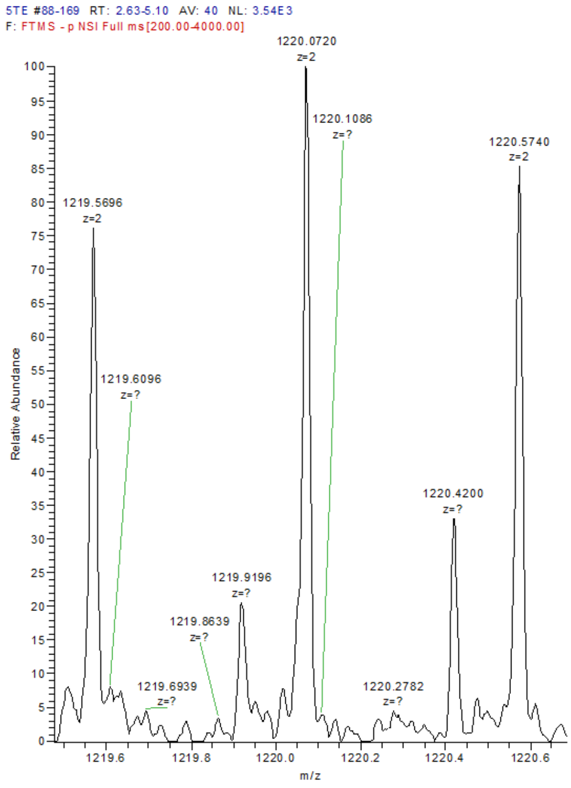
5TE #88-169 RT: 2.63-5.10 AV: 40 NL: 3.54E3
F: FTMS - p NSI Full ms [200.00-4000.00]
1219.5696 z=2
1219.6096 z=?
1219.6939 z=?
1219.8639 z=?
1219.9196 z=?
1220.0720 z=2
1220.1086 z=?
1220.2782 z=?
1220.4200 z=?
1220.5740 z=2
Relative Abundance
m/z

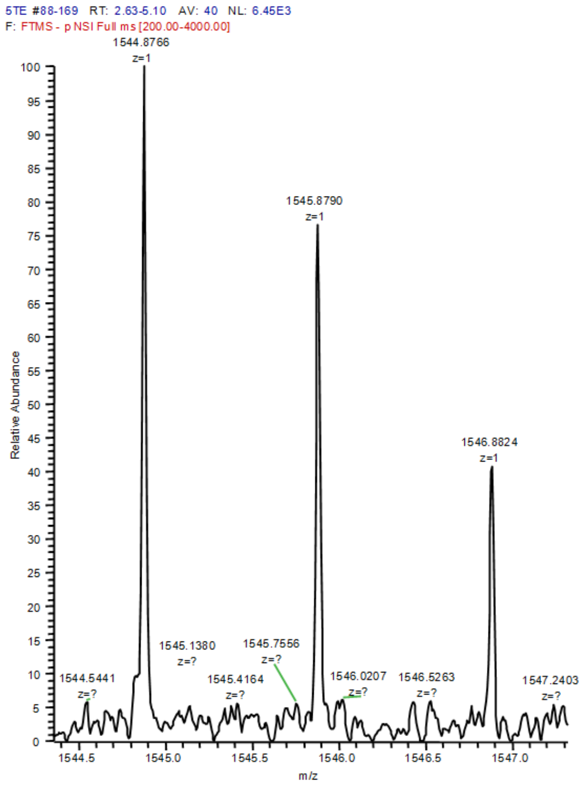
5TE #88-169 RT: 2.63-5.10 AV: 40 NL: 6.45E3
F: FTMS - p NSI Full ms [200.00-4000.00]
1544.5441 z=?
1544.8766 z=1
1545.1380 z=?
1545.4164 z=?
1545.7556 z=?
1545.8790 z=1
1546.0207 z=?
1546.5263 z=?
1546.8824 z=1
1547.2403 z=?
Relative Abundance
m/z

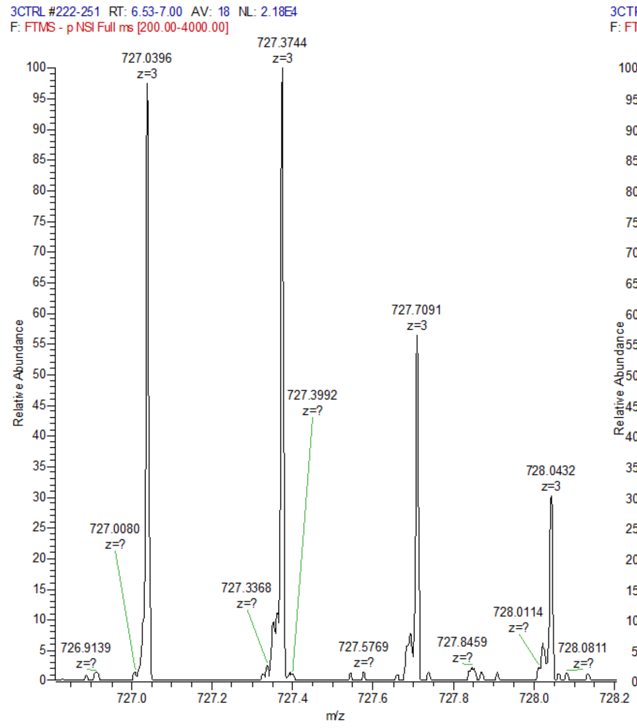
3CTRL #222-251 RT: 6.53-7.00 AV: 18 NL: 2.18E4
F: FTMS - p NSI Full ms [200.00-4000.00]
727.0396 z=3
727.3744 z=3
727.7091 z=3
727.3992 z=?
728.0432 z=3
727.0080 z=?
727.3368 z=?
728.0114 z=?
726.9139 z=?
727.5769 z=?
727.8459 z=?
728.0811 z=?
Relative Abundance
m/z

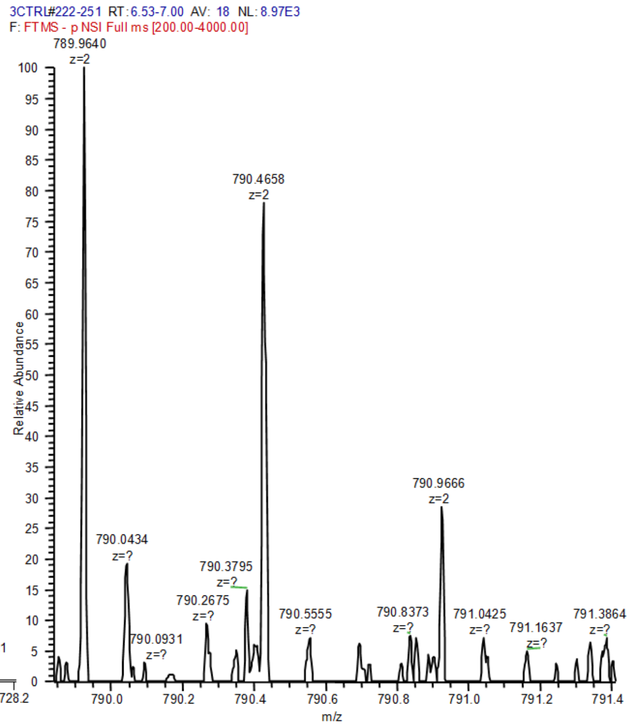
3CTRL#222-251 RT: 6.53-7.00 AV: 18 NL: 8.97E3
F: FTMS - p NSI Full ms [200.00-4000.00]
789.9640 z=2
790.4658 z=2
790.9666 z=2
790.0434 z=?
790.3795 z=?
790.2675 z=?
790.5555 z=?
790.8373 z=?
791.0425 z=?
791.3864 z=?
791.1637 z=?
790.0931 z=?
Relative Abundance
m/z

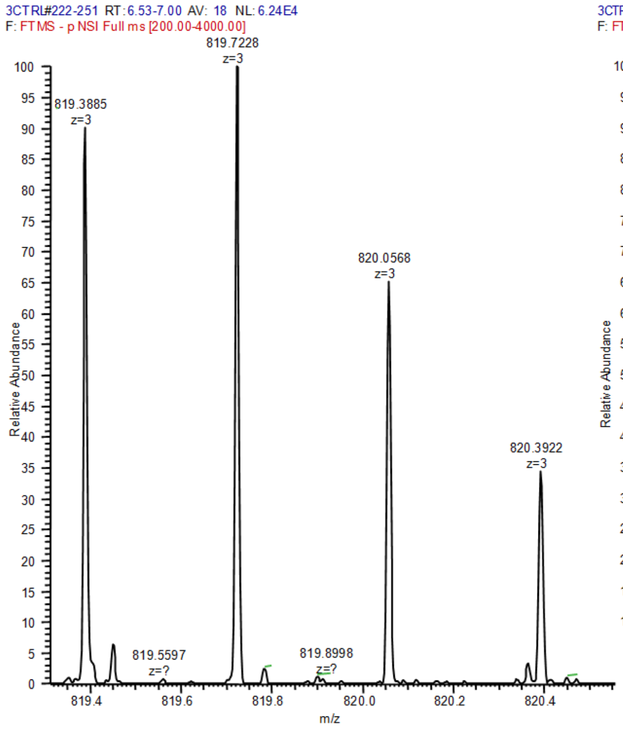
3CTRL#222-251 RT: 6.53-7.00 AV: 18 NL: 6.24E4
F: FTMS - p NSI Full ms [200.00-4000.00]
819.7228 z=3
819.3885 z=3
820.0568 z=3
820.3922 z=3
819.5597 z=?
819.8998 z=?
Relative Abundance
m/z

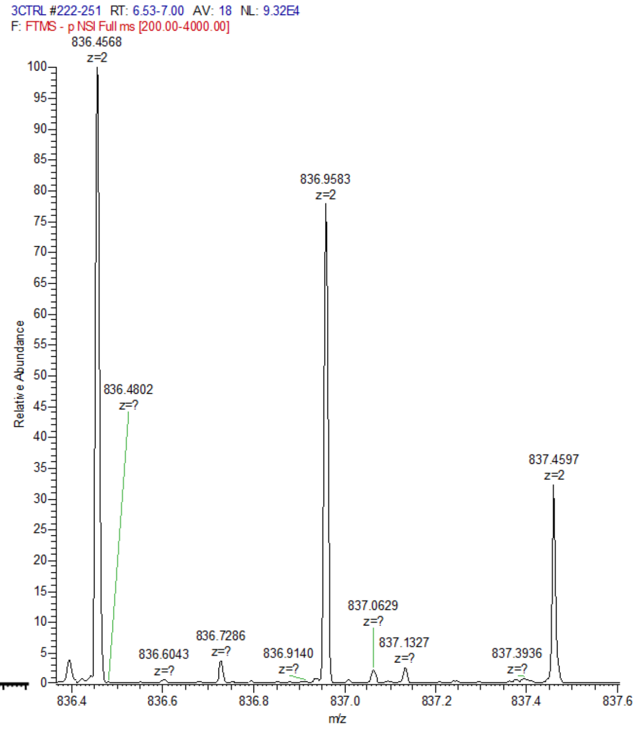
3CTRL #222-251 RT: 6.53-7.00 AV: 18 NL: 9.32E4
F: FTMS - p NSI Full ms [200.00-4000.00]
836.4568 z=2
836.9583 z=2
836.4802 z=?
837.4597 z=2
837.0629 z=?
836.7286 z=?
837.1327 z=?
836.6043 z=?
836.9140 z=?
837.3936 z=?
Relative Abundance
m/z

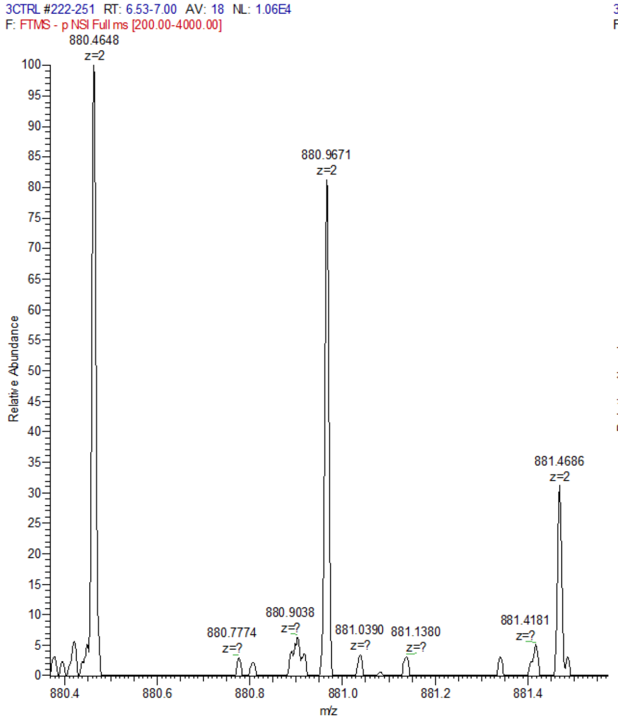
3CTRL #222-251 RT: 6.53-7.00 AV: 18 NL: 1.06E4
F: FTMS - p NSI Full ms [200.00-4000.00]
880.4648 z=2
880.9671 z=2
881.4686 z=2
880.7774 z=?
880.9038 z=?
881.0390 z=?
881.1380 z=?
881.4181 z=?
Relative Abundance
m/z

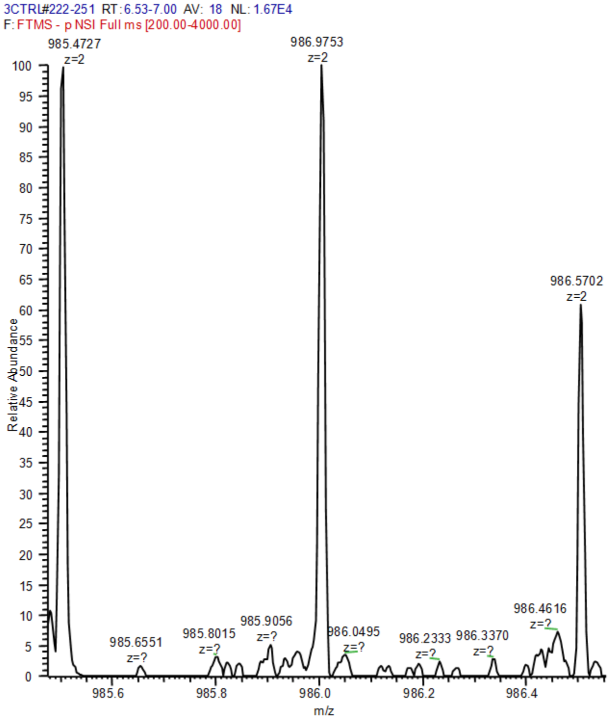
3CTRL#222-251 RT: 6.53-7.00 AV: 18 NL: 1.67E4
F: FTMS - p NSI Full ms [200.00-4000.00]
985.4727 z=2
986.9753 z=2
986.5702 z=2
985.6551 z=?
985.8015 z=?
985.9056 z=?
986.0495 z=?
986.2333 z=?
986.3370 z=?
986.4616 z=?
Relative Abundance
m/z

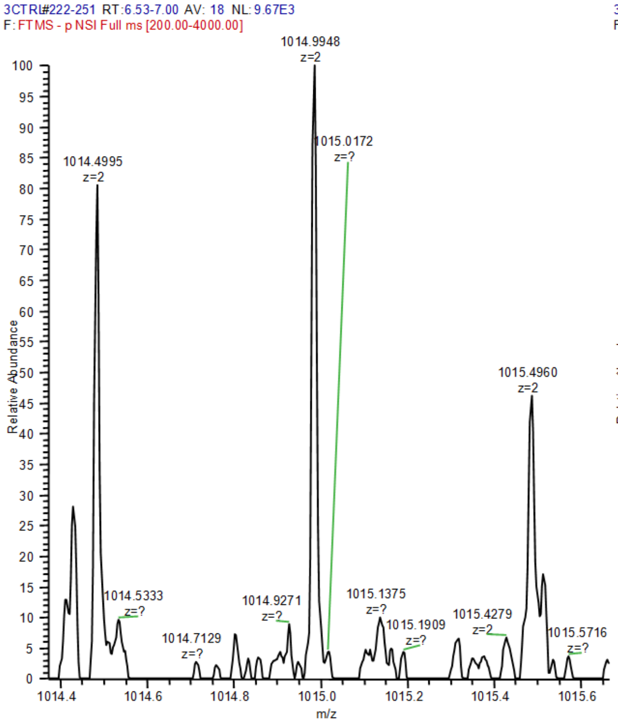
3CTRL#222-251 RT: 6.53-7.00 AV: 18 NL: 9.67E3
F: FTMS - p NSI Full ms [200.00-4000.00]
1014.9948 z=2
1015.0172 z=?
1014.4995 z=2
1015.4960 z=2
1014.5333 z=?
1014.7129 z=?
1014.9271 z=?
1015.1375 z=?
1015.1909 z=?
1015.4279 z=2
1015.5716 z=?
Relative Abundance
m/z

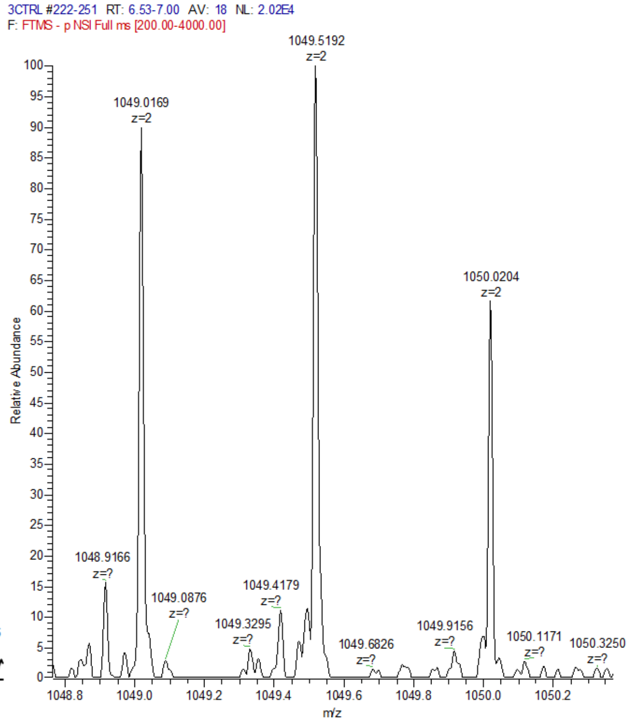
3CTRL #222-251 RT: 6.53-7.00 AV: 18 NL: 2.02E4
F: FTMS - p NSI Full ms [200.00-4000.00]
1049.5192 z=2
1049.0169 z=2
1050.0204 z=2
1048.9166 z=?
1049.0876 z=?
1049.4179 z=?
1049.3295 z=?
1049.6826 z=?
1049.9156 z=?
1050.1171 z=?
1050.3250 z=?
Relative Abundance
m/z

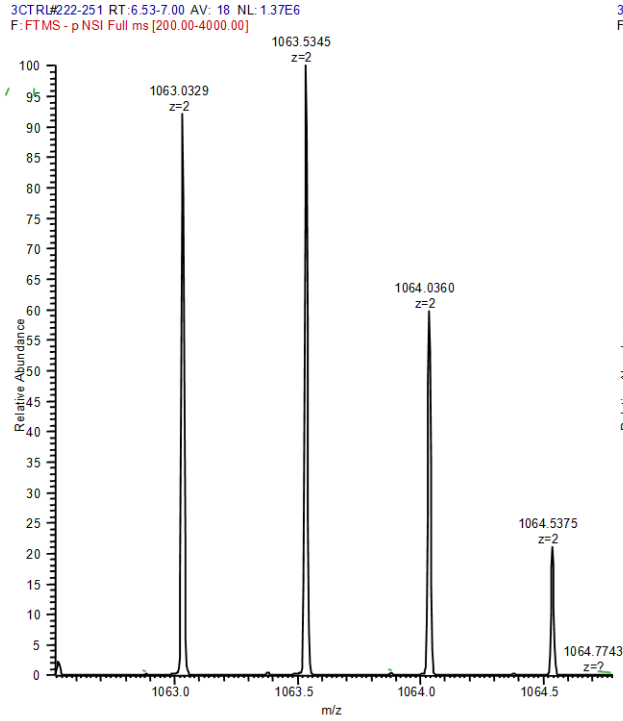
3CTRL#222-251 RT:6.53-7.00 AV: 18 NL: 1.37E6
F: FTMS - p NSI Full ms [200.00-4000.00]
1063.0329 z=2
1063.5345 z=2
1064.0360 z=2
1064.5375 z=2
1064.7743 z=?
Relative Abundance
m/z

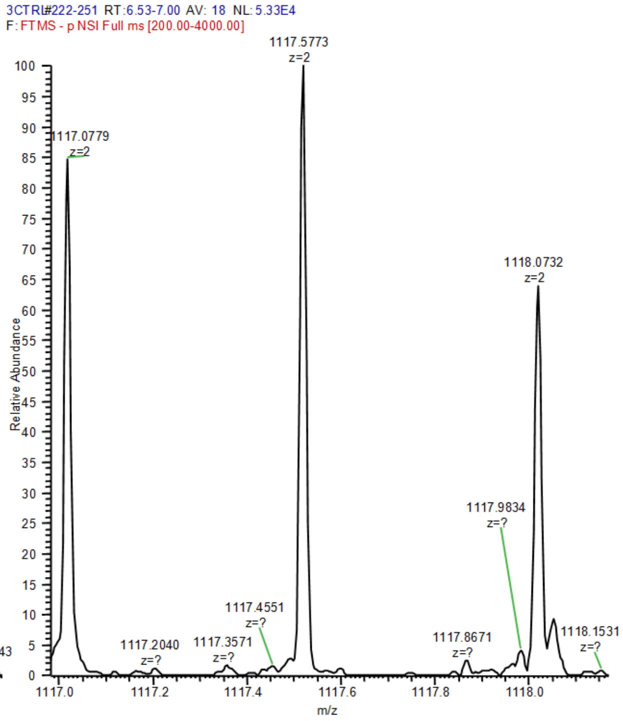
3CTRL#222-251 RT:6.53-7.00 AV: 18 NL: 5.33E4
F: FTMS - p NSI Full ms [200.00-4000.00]
1117.0779 z=2
1117.5773 z=2
1118.0732 z=2
1117.2040 z=?
1117.3571 z=?
1117.4551 z=?
1117.8671 z=?
1117.9834 z=?
1118.1531 z=?
Relative Abundance
m/z
